# Suppression of a Subset of Interferon-Induced Genes by Human Papillomavirus Type 16 E7 via a Cyclin Dependent Kinase 8-Dependent Mechanism

**DOI:** 10.3390/v12030311

**Published:** 2020-03-13

**Authors:** Sadie Rice, Seong-man Kim, Cynthia Rodriguez, William Songock, Gaurav Raikhy, Rebecca Lopez, Lauren Henderson, Arjun Yusufji, Jason Bodily

**Affiliations:** 1 Department of Microbiology and Immunology, Center for Molecular and Tumor Virology, and Feist-Weiller Cancer Center, Louisiana State University Health Sciences Center, Shreveport, LA 71103, USA; srice3@lsuhsc.edu (S.R.); sethkim70@gmail.com (S.-m.K.); cynthiarodriguez@wustl.edu (C.R.); wsongock@gmail.com (W.S.); graikh@lsuhsc.edu (G.R.); rlope4@lsuhsc.edu (R.L.); hendersonl91@lsus.edu (L.H.); ayusuf@lsuhsc.edu (A.Y.); 2 C.E. Byrd High School, Shreveport, LA 71104, USA

**Keywords:** STAT1, IFN signaling, interferon-stimulated genes, transcription, Mediator kinase CDK8, papillomaviruses, oncoprotein E7

## Abstract

Persistent infection by human papillomaviruses (HPVs), small, double-stranded DNA viruses that infect keratinocytes of the squamous epithelia, can lead to the development of cervical and other cancers. The viral oncoprotein E7 contributes to viral persistence in part by regulating host gene expression through binding host transcriptional regulators, although mechanisms responsible for E7-mediated transcriptional regulation are incompletely understood. Type I IFN signaling promotes the expression of anti-viral genes, called interferon-stimulated genes (ISGs), through the phosphorylation and activation of STAT1. In this study, we have observed that the CR3 domain of E7 contributes to the episomal maintenance of viral genomes. Transcriptome analysis revealed that E7 transcriptionally suppresses a subset of ISGs but not through regulation of STAT1 activation. Instead, we discovered that E7 associates with Mediator kinase CDK8 and this is correlated with the recruitment of CDK8 to ISG promoters and reduced ISG expression. E7 fails to suppress ISGs in the absence of CDK8, indicating that CDK8 function contributes to the suppression of ISGs by E7. Altogether, E7/CDK8 association may be a novel mechanism by which E7 inhibits innate immune signaling.

## 1. Introduction

Infection by human papillomaviruses (HPVs), small, double-stranded DNA viruses that infect keratinocytes of stratified squamous epithelia, can lead to the development of benign lesions and anogenital and oropharyngeal cancers [1,2]. Infection by certain HPV types (so-called “high risk” types) causes essentially all cervical cancer worldwide, with over half of cases caused by HPV type 16 [3]. The HPV life cycle is tightly regulated by cellular differentiation [4]. In basal epithelial cells, viral genomes are maintained as episomes at a low copy number. As the host cell detaches from the basal layer and undergoes terminal differentiation, the viral late promoter is activated to drive the productive phase of infection in which genomes are replicated to high copy numbers, capsid proteins are synthesized, and progeny virions are assembled and released [4,5,6]. This life cycle organization is one mechanism by which the virus persists as it restricts the production of antigenic viral proteins to the upper, differentiated layers of the skin that have reduced immune-surveillance [7,8,9,10]. Persistent infection by high risk HPV types is the major risk factor for the development of cancer as this allows for genetic mutations to accumulate in proliferating, infected cells [1]. 

In addition to its overall life cycle organization, HPV16 encodes several oncoproteins that function to promote the viral life cycle and are required for viral persistence. The oncoprotein E7 is the main transforming protein and is sufficient to immortalize human epithelial cells [11,12,13]. E7 contributes to viral persistence, in part, by promoting cell cycle progression and inhibiting host immune responses [14,15]. Interactions between the three conserved domains of E7 (CR1-3) and numerous transcriptional regulators have been reported [14,16], including with hypoxia-inducible factor 1 (HIF1α) [17], chromatin modifying enzymes [18,19,20], TATA box binding protein (TBP) [21], interferon regulatory factor 1 (IRF1) [22], and transcriptional repressor E2F6 [23]. E7 is best known for binding to and targeting the transcriptional repressor retinoblastoma (pRb) for proteasomal degradation to promote cell cycle progression in differentiated keratinocytes, which facilitates the replication of viral genomes [24,25,26].

Type I interferons (IFNs), which include IFN-α and IFN-β, signal through the Janus kinase/signal transducer and activator of transcription (JAK/STAT) pathway. Importantly, human foreskin keratinocytes (HFKs) constitutively produce IFN-κ, a keratinocyte-specific IFN that stimulates type I IFN signaling [27]. Autocrine or paracrine IFN stimulation induces STAT1 and STAT2 to dimerize, bind IRF9 to form the IFN-stimulated gene factor 3 (ISGF3) complex, and translocate to the nucleus to bind the interferon-specific response element (ISRE) of target promoters [28,29,30]. Nuclear STAT1 gains full transcriptional activity once phosphorylated in the carboxy-terminal transactivation domain on serine 727 (pS727) to drive the expression of genes induced by both type I and type II IFNs [31,32,33,34,35,36]. These IFN-stimulated genes (ISGs) have a variety of anti-viral activities [37]. It has been reported that E7 can inhibit IFN signaling by binding IRF9 to block formation of the ISGF3 complex and prevent nuclear translocation [38], along with other potential mechanisms [22,39,40,41,42,43,44]. While many E7 interacting partners have been reported, our understanding of the biological significance of these interactions remains largely incomplete. Numerous studies have reported that certain CR3 mutations can alter E7’s biological activities [14,17,45,46,47], but, because E7 is indispensable for completion of the HPV life cycle, generation of HPV16+ cells harboring mutations in the E7 open reading frame (ORF) has been largely unsuccessful using primary keratinocytes. A study by Todorovic et al. used the HPV1A E7 CR3 domain crystal structure as a guide to identify CR3 residues that likely affect HPV16 E7’s ability to interact with cellular proteins without disrupting the overall structure of the CR3 domain [47]. 

Cyclin-dependent kinase 8 (CDK8) is a regulator of transcriptional complexes and is a component of the CDK8 submodule of the Mediator complex [48]. CDK8 kinase activity can affect transcription through positive or negative regulation of certain activators and transcription factors and the phosphorylation of histone H3S10 [49,50]. The CDK8 submodule also has kinase-independent regulatory functions, such as regulation of the association of RNA polymerase II (Pol II) with Mediator [51], the recruitment of factors required for transcriptional elongation [52,53], and histone H3K9 methylation [54]. 

This paper is part of a larger effort to investigate the consequences of E7 CR3 mutations in order to uncover novel biological activities of E7. We report that certain residues in the CR3 domain of E7 are critical for episomal maintenance of the viral genome. The CR3 domain influences the ability of E7 to suppress a subset of type I IFN-induced ISGs. We found E7 interacts with CDK8 and that E7 requires CDK8 to suppress ISG expression. Additionally, CDK8 is enriched at ISG promoters in cells containing E7 but there is reduced CDK8 enrichment in cells containing the E7 F57A mutant, suggesting that E7 regulates CDK8 occupancy at ISG promoters. Altogether, our data suggest a novel function by which E7 suppresses IFN signaling in a manner that requires interacting with and altering CDK8 function.

## 2. Materials and Methods

### 2.1. Cloning of E7 Mutants

HPV16 genomes containing CR3 mutations in the E7 open reading frame were created by mutagenesis of wild type pEGFP Ni HPV16 plasmid with the QuickChange II Site Directed Mutagenesis kit (Agilent, Santa Clara, USA) using the primers listed in Supplementary Table S1. Mutagenesis was based on the work of Todorovic et al. [47] in which surface exposed amino acid residues in CR3 domain were replaced with residues of opposite charge. The presence of the mutations was confirmed by sequencing. To create expression vectors for E7 mutants, mutagenized pEGFP Ni HPV16 plasmids were used as templates for PCR using 16E7 Xho frame 5’ and 16E7 Not stop 3’ (Supplementary Table S1). These fragments were digested with XhoI and NotI and cloned into digested pcDNA TapN 16E7 [17]. The E7 CR2 LYCYE deletion mutant was created as previously described [45]. The pLXSN E6/E7 F57A plasmid was created by site directed mutagenesis using the primers in Supplementary Table S1. 

### 2.2. Cell Culture and Creation of Cell Lines

Human foreskin keratinocytes (HFKs) were isolated from discarded and de-identified neonatal foreskins; HFKs containing HPV16 genomes (W12 strain) were created by transfection and selection as previously described [45]. Episomal maintenance of the virus was confirmed by Southern analysis: total DNAs were isolated and digested with XhoI (which does not cut the HPV16 genome) before being analyzed by Southern blotting using the whole HPV16 genome as a probe as described previously [45]. HFKs expressing HPV oncogenes were created by retroviral transduction as described previously [45,55]. Retroviral stocks were generated by transfection of retrovirus vector plasmids and into a packaging cell line as previously described [56]. HFKs and keratinocyte-derived cell lines were cultivated in E medium with 5% fetal bovine serum (FBS) (HyClone, Logan, USA) in the presence of mitomycin C-treated NIH-3T3 J2 fibroblast feeders [45,57]. Cell lines derived from at least three donors were used in separate experiments, and data for all figures were compiled from at least three individual experiments. U2OS cells were cultivated in DMEM (Gibco, Grand Island, USA) containing 10% bovine growth serum (BGS) (HyClone, Logan, USA).

The drugs used in this study are Ruxolitinib (Selleckchem, Houston, USA, #S1378), Senexin A (Tocris, Bristol, United Kingdom, #4875), and recombinant IFNβ (PBL Assay Science, Piscataway, USA, #11415-1). Drugs were reconstituted per manufacturer’s instructions and added to monolayer media at the time of seeding at the following concentrations: 10 μM of Ruxolitinib, 10 μM of Senexin A, or 50 units/mL of IFNβ.

### 2.3. Cellular Growth Rates

To determine cellular growth rate, each cell line was cultured in a monolayer, passaged at a 1:10 dilution. The number of days required for cells to reach confluency after each passage (i.e., time required for 3.3 population doublings) was recorded for a maximum of 60 days.

### 2.4. RNA Extraction, RT-qPCR, and Western Blotting

Cells were seeded and grown in monolayer for 24 h and total RNA was isolated using RNA-STAT 60 (TelTest, Inc. Friendswood, USA), digested with RNase-free DNase (Promega, Madison, USA), phenol-chloroform extracted, and reverse transcribed using qScript (Quanta, Beverly, USA). Quantitative PCR (qPCR) was performed using the PerfeCTa SYBR green SuperMix ROX (Quanta, Beverly, USA) on an Applied Biosystems StepOne Plus real-time PCR machine using the primers listed in Table S1. Western blotting was performed as follows: Cells were grown in monolayer for 24 h and protein lysates were prepared by adding 1× Lysis Buffer (Cell Signaling, Danvers, USA, supplemented with 1 mM PMSF) to cells and incubated on ice for 5 min followed by scraping, brief sonication, and clarification by centrifugation. SDS-PAGE and Western blotting were performed as described previously [56] with 100 μg of protein. Blocking and antibody dilution was performed using Li-Cor Odyssey® blocking buffer containing 0.1% tween-20 and images were acquired using a Li-Cor Odyssey® near infrared imaging system. Antibodies used include: CDK8 (Bethyl Laboratories, Montgomery, USA, #A302-501A), pRb (Cell Signaling, Danvers, USA, #9309S), p53 (Calbiochem, San Diego, USA, #OP43), phospho-STAT1 (Y701; Cell Signaling, Danvers, USA, #9167), phospho-STAT1 (S727; Cell Signaling, Danvers, USA, #8826), total STAT1 (Cell Signaling, Danvers, USA, #9172), GAPDH (Santa Cruz Biotechnology, Dallas, USA, #47724), and HPV16 E7 (Valdospan GmbH, Tulln, Austria, #VS13004L). Band intensity was quantified using Image Studio Lite Software (Li-Cor, Lincoln, USA).

### 2.5. RNA Sequencing

Total RNA was isolated using RNA-STAT 60 (TelTest, Inc., Friendswood, USA), and purified using the RNeasy RNA isolation kit (Qiagen, Hilden, Germany). Total RNA integrity was assessed on an Agilent TapeStation 2200 using RNA ScreenTape assay. Libraries were prepared using Illumina’s TruSeq Stranded RNA LT kit. Libraries were analyzed on an Agilent TapeStation 2200 D1000 assay to determine average size and were quantitated using the NEBNext Library Quant qPCR Kit. Libraries were normalized to 4 nM, pooled, denatured, and diluted to approximately 1.8 pM. A 1% library of 1.8 pM PhiX was spiked in as an internal control. The library pool was sequenced on an Illumina NextSeq 550, with a read length of 101 × 51 base pairs. Base calling and quality scoring were performed with Illumina Real Time Analysis software (RTA). Two runs were performed. Reads were aligned to the human (GRCh38.p10) and HPV16 (NC_001526.3) genomes using STAR_2.4.2a and counted using RSEM 1.2.31. Differentially expressed genes were identified with EBSeq 1.12. A gene was considered differentially expressed if expression was at least 2-fold change different in pLXSN E6/E7 F57A cells, as compared to pLXSN E6/E7 cells, and had a *p* value of 0.05 or less.

All pathway analyses were performed using Reactome [58,59]. Reactome performs a statistical (hypergeometric distribution) test that determines whether certain Reactome pathways are over-represented in a list of genes [58,59]. The lists of genes submitted to Reactome consisted of all up- or downregulated genes in pLXSN E6/E7 F57A cells, as compared to pLXSN E6/E7 cells. Significantly enriched pathways were determined by a false discovery rate (FDR) of 0.05 or less. The FDR is the corrected over-representation probability calculated using the Benjamini-Hochberg approach [58,59]. Reactome results were reported in table format. Entities found refers to the different components of the pathway that correspond to the submitted genes (the up- or downregulated list of genes). A gene may map to more than one entity in a certain pathway, as it may represent the gene, protein, or a modified protein within the listed pathway. Entities total refers to all the components within the listed pathway [58,59].

### 2.6. siRNA Transfection

CDK8 was targeted with ON-TARGETplus SMARTpool L-003242-00-0005 and the negative control was D-001810-10-20 (both from Dharmacon, Lafayette, USA). The DharmaFECT siRNA protocol was followed. Briefly, cell lines were seeded in a 6-well plate at 500,000 cells/well and incubated with E medium + 5% FBS. Twenty-four hours post seeding, the siRNA was diluted to 35 nM in Opti-MEM (Gibco, Grand Island, USA, #11058-021) using DharmaFECT1 (Dharmacon, Lafayette, USA, T-2001-02) at a concentration of 5 μL/mL and added to cells incubated with E medium + 5% FBS, according to the manufacturer’s protocol. Cells were harvested for RNA or protein as described above 72 h post-transfection.

### 2.7. Immunoprecipitation

U2OS cells were transfected with 1 μg of HA-tagged E7 expression plasmid overnight using polyethyleneimine (PEI; Polysciences, Warrington, USA). Immunoprecipitation was performed as described previously using the anti-CDK8 antibody (Abcam, Cambridge, United Kingdom, ab176559) for immunoprecipitation and anti-HA antibody (Santa Cruz Biotechnology, Dallas, USA, sc-7392) for Western blotting and detection of HA-E7 [17]. From HPV16+ cells, HPV E7 and CDK8 were immunoprecipitated following the manufacturer’s instructions in the Pierce Cross-link IP kit (Thermo Fisher Scientific, Waltham, USA, #26147). Briefly, based on primary antibody source 20 μL of either protein A or protein G agarose per sample was added to a spin column and washed with IP lysis/wash buffer (25 mM Tris-HCl (pH 7.4), 150 mM NaCl, 1 mM EDTA, 1% Igepal CA-630, 5% glycerol). Two micrograms of either E7 (Valdospan GmbH, Tulln, Austria, #VS13004L) or CDK8 (Bethyl Laboratories, Montgomery, USA, A302-500A) antibody per sample was added to the prewashed protein A or G agarose and incubated at RT for 1 h. The primary antibody was cross-linked to the protein A or G agarose by adding the cross-linking reagent disuccinimidyl suberate (DSS) to a final concentration of 25 mM and incubating at RT for 1 h. Anti-CDK8-crosslinked protein A agarose or anti-E7-crosslinked protein G was washed twice with elution buffer (50 mM glycine (pH 2.8)) to remove non-cross-linked antibody and to quench the reaction followed by equilibration with IP lysis/wash buffer. Pellets of wild type HPV16-containing HFKs were resuspended in 500 μL of IP lysis/wash buffer containing protease inhibitor cocktail (Thermo Fisher Scientific, Waltham, USA). The cell lysates were centrifuged at 13,000× *g* for 10 min at 4 °C; the lysate was transferred to new tube and protein concentration was measured with Bradford’s assay. One thousand and five hundred micrograms of whole cell lysate were transferred to a new tube, and 20 μL of cross-linked antibody-agarose was added, followed by gentle rocking overnight at 4 °C. The complex was washed 2× with IP lysis/wash buffer and once with 50 mM HEPES, pH 7. The bound proteins were eluted by the addition of 50 μL of 1× SDS sample buffer and boiling at 95 °C. 

### 2.8. Chromatin Immunoprecipitation

Cells grown in monolayer culture were trypsinized and resuspended in 10 mL E medium. Formaldehyde was added to a final concentration of 1% and incubated for 15 min at room temperature with rocking. Next, 1 mL of 1.25 M glycine was added and incubated for an additional 5 min at room temperature. Cells were then washed three times with ice cold PBS containing protease inhibitors and resuspended in 1× Lysis Buffer (Cell Signaling, Danvers, USA) at a final cell density of 10 million cells/mL. ChIP was performed by coupling 3 μg of antibody to Protein G Dynabeads (Invitrogen, Carlsbad, USA) and incubating with 5 × 10^6^ cells. Dynabeads were washed and suspended in 5 mg/mL BSA buffer and then incubated with IgG (Santa Cruz Biotechnology, Dallas, USA, #2027) or CDK8 (Bethyl Laboratories, Montgomery, USA #A302-500A) antibodies overnight at 4 °C with rotation. The following day, cells were sonicated briefly and then treated with micrococcal nuclease (final concentration 60 U/μL, New England Biolabs, Ipswich, USA) for 1 h on ice. EDTA was added to a final concentration of 50 mM and debris was removed by centrifugation. Dilution buffer (1% Triton X-100, 0.1% deoxycholate (DOC) sodium salt, 1 mM PMSF, TE) was added to chromatin and Dynabeads blocked with 5 mg/mL BSA buffer were added to chromatin to pre-clear for 1 hour at 4 °C with rotation. The antibody Dynabead complexes incubated overnight were washed three times with 1 mL of 5 mg/mL BSA buffer and suspended in 100 μL of 5 mg/mL BSA buffer. Following pre-clearing, the antibody Dynabead complexes were added to chromatin and incubated overnight at 4 °C with rotation. Beads were washed seven times in freshly prepared RIPA buffer (50 mM HEPES pH 8.0, 1mM EDTA, 1% NP-40, 0.7% DOC sodium salt, 0.5 M LiCl, 1 mM PMSF, dH_2_O) and once with TE buffer (Fisher) by gently inversion. Beads were resuspended in TE buffer containing 0.3% SDS, 200 mM NaCl, and 0.5 mg/mL proteinase K (Sigma, St. Louis, USA) and incubated for 2 h at 45 °C followed by 65 °C overnight. Supernatants were collected, beads were washed with TE buffer containing 500 mM NaCl, and wash was added to the supernatants. DNA was purified using the PCR Clean-up DNA Purification Kit (MoBio, Hilden, Germany). Immunoprecipitated DNA fragments were subjected to qPCR as described above using the primers listed in Supplementary Table S1.

### 2.9. Statistics

RT-qPCR analysis included at least 3 technical replicates and *n* ≥ 3 biological replicates using cell lines derived from separate foreskin keratinocyte donors. Immunoblot densitometry analysis included at least 1 technical replicate of *n* ≥ 3 biological replicates using cell lines derived from separate foreskin keratinocyte donors. The significance of genome integration rates between wild type HPV16 and HPV16 CR3 mutants was determined using Fisher’s exact test. Significance of other experiments was calculated using Welch’s unequal-variance t test. *p* values are as follows: * = *p* < 0.05, ** = *p* < 0.01, *** = *p* < 0.001.

## 3. Results

### 3.1. The CR3 of E7 Contributes to Episomal Maintenance of the HPV16 Genome

The CR3 domain is comprised of amino acids 38–98 of the E7 protein. CR3 contains two cysteine motifs that facilitate zinc binding and formation of the tertiary protein structure, and is required for transformation [47,60]. Certain mutations of the hydrophobic core residues of CR3, such as L67R, are reported to disrupt E7’s association with host factors but this may be due to significant structural changes of the C terminus rather than disruption of specific binding residues on E7 [14,47]. Surface-exposed residues that are accessible to mediate interaction with cellular proteins but which are not needed to maintain the overall tertiary structure have been modeled for HPV16 E7 based on the x-ray crystal structure of the CR3 domain of HPV1A [47,61]. We sought to determine how CR3 mutations affect cellular immortalization, growth rate and episomal maintenance of viral genomes. Using human foreskin keratinocytes (HFKs), we created cell lines that contain the complete wild type HPV16 genome (HPV16+ cells), or HPV16 genomes harboring single amino acid mutations in the CR3. In total, we generated thirteen cell lines that harbor unique CR3 mutations (Y52A, N53D, M84S, G85A, D62K, ED80-81KK, S63D, V55T, QKP96-98EEA, F57A, R66E, T64D, R77E) in at least three HFK donor backgrounds. HFKs containing HPV null E7 genomes were not generated because E7 is required for immortalization, as noted by us and others [11,45]. Cellular immortalization was not disrupted by any of the tested CR3 mutations. There was no significant difference in cellular growth rate between wild type HPV16+ cells and cells containing CR3 mutant genomes (Figure S1a). In preliminary studies, CR3 cell lines were analyzed by Western blot for E7 and p53 (surrogate for E6) but there were no consistent differences in protein levels when compared to HPV16+ cells, and therefore was not investigated further. Southern blotting was used to determine how CR3 mutations affect long-term episomal maintenance of viral genomes. Table 1 shows the total number of samples for each cell line in which the cell population was either episomal or integrated. Seven mutant genomes (S63D, V55T, QKP96-98EEA, F57A, R66E, T64D, R77E) integrated at a significantly higher frequency than wild type genomes (*p* < 0.05) (Table 1). Together, these data demonstrate that the CR3 of E7 contributes to proper episomal maintenance of the HPV16 genome. 

### 3.2. E7 F57A Is Defective in E7-Mediated Suppression of ISGs

Previous work has demonstrated that type I IFN signaling promotes viral genome integration [56,62,63,64]. We sought to investigate whether CR3 mutations associated with integration alter the expression of genes involved in IFN signaling or other immune-related pathways, as compared to wild type E7. The F57A mutant was selected for further analysis based on the abundance of Southern blot data that show frequent integration of F57A genomes (Table 1). Directly comparing the gene expressing profiles of cells containing the F57A mutant to wild type HPV16 was problematic due to the fact that the F57A genome was integrated into the host genome, thus potentially changing the pattern of gene expression. 

**Figure 1:**
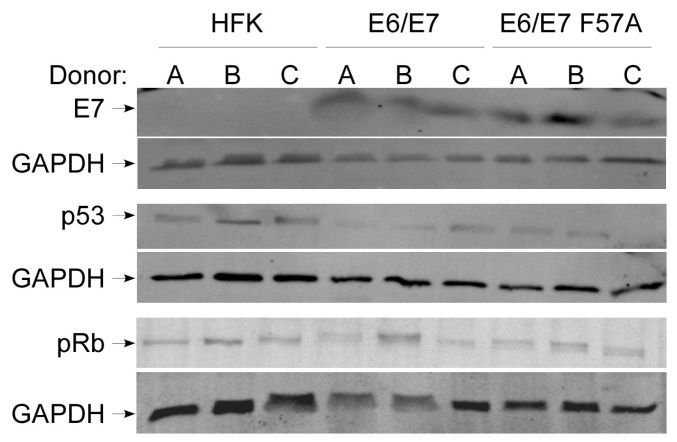
Characterization of E6/E7 and E6/E7 F57A cell lines. Western blot analysis of protein levels of E7 (top panel), p53 (middle panel), pRb (bottom panel), and glyceraldehyde-3-phosphate dehydrogenase (GAPDH) in human foreskin keratinocytes (HFKs), pLXSN E6/E7, and pLXSN E6/E7 F57A cells grown in monolayer culture. Three HFK donors (**A**–**C**) were used.

To test our hypothesis and circumvent the problem of genome integration, we generated cell lines immortalized with E6 and E7 (pLXSN E6/E7 cells) or E6 and E7 F57A (pLXSN E6/E7 F57A cells) expressed from retroviral vectors. Our successful generation of immortalized cells containing E7 F57A is in agreement with the findings of Todorovic et al. who showed this mutation does not affect the ability of E7 to transform primary baby rat kidney (BRK) cells [47], and also with the ability of F57A-containing genomes to immortalize primary keratinocytes (above). Western blot showed that the E7 F57A mutation did not negatively affect E7 levels, as there was no difference in E7 levels between E6/E7 and E6/E7 F57A cells (Figure 1, top panel). Data also suggested that the structure of E7 is not grossly altered as the antibody detected E7 F57A. As a surrogate for the activity of E6, we compared total p53 levels between pLXSN cell lines by Western blot. There was no apparent difference in p53 levels between E6/E7 and E6/E7 F57A cell lines indicating E6 functions similarly in these cells (Figure 1, middle panel). As a measure of the pRb degradation activity of E7, we performed Western blot for pRb. There was no consistent difference in pRb levels between E6/E7 and E6/E7 F57A cell lines indicating the E7 F57A mutation did not alter the ability of E7 to promote pRb degradation, which further supports our data and other’s that the F57A mutation does not decrease the transformation ability of E7 (Figure 1, bottom panel) [47]. 

We next sought to identify genes whose expression may be modified in the presence of the mutant vs. wild type E7 using RNA-sequencing with total RNA extracted from E6/E7 and E6/E7 F57A cells. A total of 25 genes were upregulated and 175 genes were downregulated by at least two fold in the E6/E7 F57A cells as compared to E6/E7 controls (File S1). Genes up- or downregulated in E6/E7 F57A cells, as compared to E6/E7 cells, were subjected to Reactome pathway analysis [58] (File S2). Analysis of the set of downregulated genes revealed no pathways as significantly enriched. In contrast, there were five pathways predicted to be significantly enriched (FDR < 0.05) based on the upregulated genes, and of these the top three were IFN- or immune-related signaling (Table 2). 

**Figure 2:**
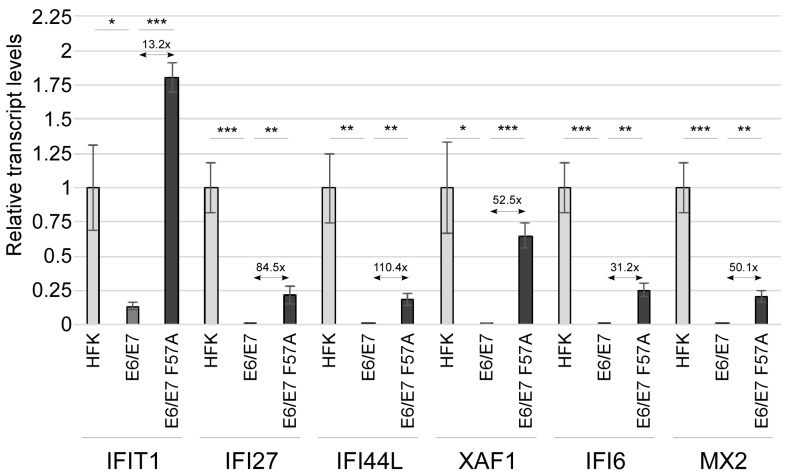
Levels of interferon-stimulated genes (ISG)s. RT-qPCR analysis for ISGs (IFIT1, IFI27, IFI44L, XAF1, IFI6, and MX2) in HFKs, pLXSN E6/E7, and pLXSN E6/E7 F57A cells grown in monolayer culture. Values were normalized to the cyclophilin A housekeeping gene, with HFK set to 1.

Examination of the upregulated genes revealed that E6/E7 F57A cells have increased expression of a subset of type I IFN-induced ISGs (BST2, IFI27, IFI44L, IFI6, MX2, and XAF1) (Table 3, ISGs are bold font). RNA-seq results were validated by RT-qPCR (Figure 2). BST2 was not consistently regulated by E7 in different HFK donor backgrounds and was excluded from further analyses. We also measued the levels of ISGs not identified by the RNA-seq and found that IFIT1 was regulated in a similar manner as the other ISGs (Figure 2), and therefore was included in subsequent experiments. ISG transcripts were found to be highest in HFKs. In comparison to HFKs, ISG transcripts were significantly reduced by wild type E6/E7, consistent with previous results showing that E6 and E7 each can suppress innate immune signaling [15,65]. However, E6/E7 F57A cells showed a partial defect in this suppression when compared to E6/E7 cells (note the fold change on graph). These data demonstrate that the C terminus of E7 contributes to the suppression of a subset of ISGs.

### 3.3. E7 May Function Downstream of JAK Activation to Suppress Interferon-Stimulated Gene Expression

**Figure 3:**
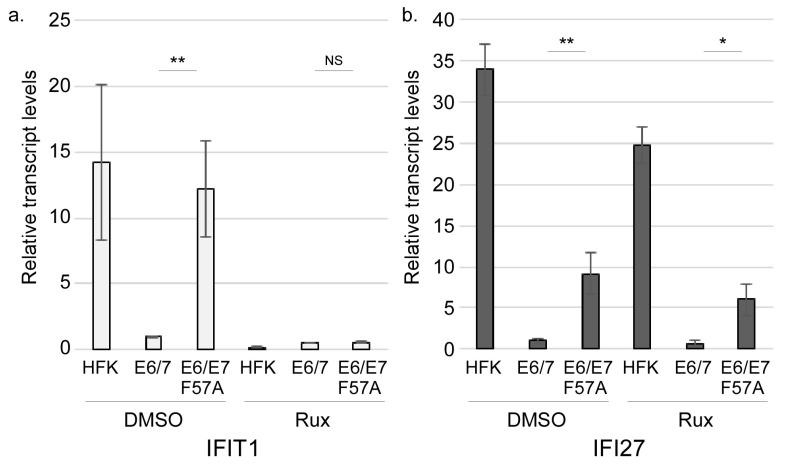
Effect of Ruxolitinib on ISG levels. RT-qPCR analysis of IFIT1 (**a**) or IFI27 (**b**) transcript levels in HFKs, pLXSN E6/E7, and pLXSN E6/E7 F57A cells treated with DMSO (control) or 10 μM Ruxolitinib (Rux) for 24 h under monolayer culture conditions. Transcripts were normalized to cyclophilin A housekeeping gene, with DMSO-treated pLXSN E6/E7 samples set to 1.

We hypothesized that E7 functions at a regulatory stage of the IFN signaling pathway to block expression of ISGs in a manner that is defective in E7 F57A. Thus, we examined key activation events of the IFN pathway for differences between E6/E7 and E6/E7 F57A cells. We first sought to determine if differences in ISG levels between E6/E7 and E6/E7 F57A were dependent on JAK-mediated phosphorylation and activation of STATs. To this end, HFKs and pLXSN cell lines were treated with DMSO or Ruxolitinib (a JAK inhibitor) for 24 h and RT-qPCR was performed for ISG transcripts. For all genes in vehicle-treated cells, there were significantly higher transcripts in E6/E7 F57A cells compared to E6/E7 cells, confirming RT-qPCR validation of the RNA-sequencing results (Figure 3, compare with Figure 2). Two different patterns of response to Ruxolitinib were observed. The first pattern included IFIT1 and MX2, in which Ruxolitinib efficiently reduced transcript levels and erased the difference between E6/E7 F57A and E6/E7 cells (Figure 3a and Figure S2a). These data suggest that the suppression defect in E6/E7 F57A cells may be due to higher activity of the JAK pathway. A second pattern was seen in all other tested ISGs (IFI27, IFI44L, IFI6, and XAF1), in which transcript levels were reduced significantly by Ruxolitinib but remained significantly higher in Ruxolitinib-treated E6/E7 F57A cells compared to Ruxolitinib-treated E6/E7 cells (Figure 3b and Figure S2b). As the suppression defect of E7 F57A persists upon JAK inhibition, we reason that E7 functions downstream of JAK activation. Interestingly, in contrast to all other ISGs tested, Ruxolitinib treatment did not reduce IFI6 expression in HFKs, as compared to vehicle-treated HFKs (Figure S2b), indicating that JAK activity is not required for IFI6 expression, supporting the hypothesis that E7 does not function to block JAK kinases.

### 3.4. E7 Does not Regulate STAT Activation

**Figure 4:**
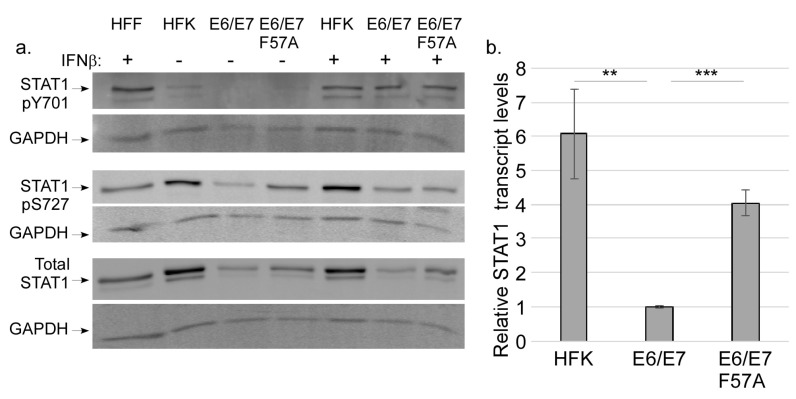
STAT1 levels and modification. (**a**) Western blot analysis of protein levels of pY701 (top panel), pS727 (middle panel), total STAT1 (bottom panel), and GAPDH in HFKs, pLXSN E6/E7, and pLXSN E6/E7 F57A cells that were treated with vehicle (IFNβ−) or 50 U/mL IFNβ (IFNβ+) for 2 h under monolayer culture conditions. Protein from uninfected human foreskin fibroblasts (HFF) treated with IFNβ was a positive control for STAT1 activation. (**b**) RT-qPCR analysis of STAT1 transcripts in untreated HFKs, pLXSN E6/E7, and pLXSN E6/E7 F57A cells. Transcripts were normalized to cyclophilin A housekeeping gene with pLXSN E6/E7 values set to 1.

During canonical type I IFN signaling, STAT1 becomes activated through phosphorylation at Y701 and S727 [33]. To determine whether E7 inhibits STAT1 activation to suppress ISGs, we first measured pY701 levels in HFK, E6/E7 and E6/E7 F57A cells. Lysate from human foreskin fibroblasts (HFF) treated with IFN-β was used as a positive control. Interestingly, we usually failed to detect pY701 in untreated cell lines; occasionally a faint band was detected in HFK or E6/E7 F57A cells (Figure 4a, top panel). We were surprised by the absence of pY701 in HFKs and E6/E7 F57A cells as they both have robust ISG expression (Figure 2). As these results were unexpected, we tested whether stimulation by an exogenous IFN could induce pY701 by treating HFK and pLXSN cell lines with IFN-β1 for 2 h. Indeed, treatment with IFN-β1 induced phosphorylation of Y701 (Figure 4a, top panel), demonstrating that the canonical STAT1 activation can occur in keratinocyte cell lines, regardless of E7 expression. Previous reports have described that loss of pY701 can occur under the condition of chronic treatment with exogenous IFN and this results in the prolonged expression of a subset of ISGs [66]. We reason that the lack of pY701 in keratinocytes could be due to the constitutive production of IFN-κ by keratinocytes, leading to chronic IFN signaling. As pY701 was not present in HFKs, these findings indicate that the phosphorylation of Y701 is not a likely point of regulation by E7.

Though Y701 is only weakly present or absent, we reasoned that nuclear STAT1 could still be regulated by S727 phosphorylation. Despite the lack of significant pY701 phosphorylation, pS727 was readily detected in all cell lines (Figure 4a, middle panel). In comparison to HFKs, E6/E7 cells had reduced levels of pS727. There was more pS727 in E6/E7 F57A cells when compared to E6/E7 cells (Figure 4a, middle panel), suggesting that E7 may suppress levels of pS727. However, evaluation of total STAT1 levels showed a pattern similar to pS727 (Figure 4a, bottom panel), indicating that the higher pS727 levels in the F57A mutant reflected overall higher levels of total STAT1. STAT1 itself is an ISG, thus higher levels of STAT1 in HFK and E6/E7 F57A cells might be expected. RT-qPCR showed that in comparison to E6/E7 cells, HFKs and E6/E7 F57A cells had significantly higher expression of STAT1 transcripts (Figure 4b), further demonstrating that E7 F57A is defective in the suppression of ISGs. Altogether, we interpret these data to suggest that STAT activation is not a point of regulation of ISGs by E7.

### 3.5. CDK8 Associates with E7 and Contributes to ISG Suppression

**Figure 5:**
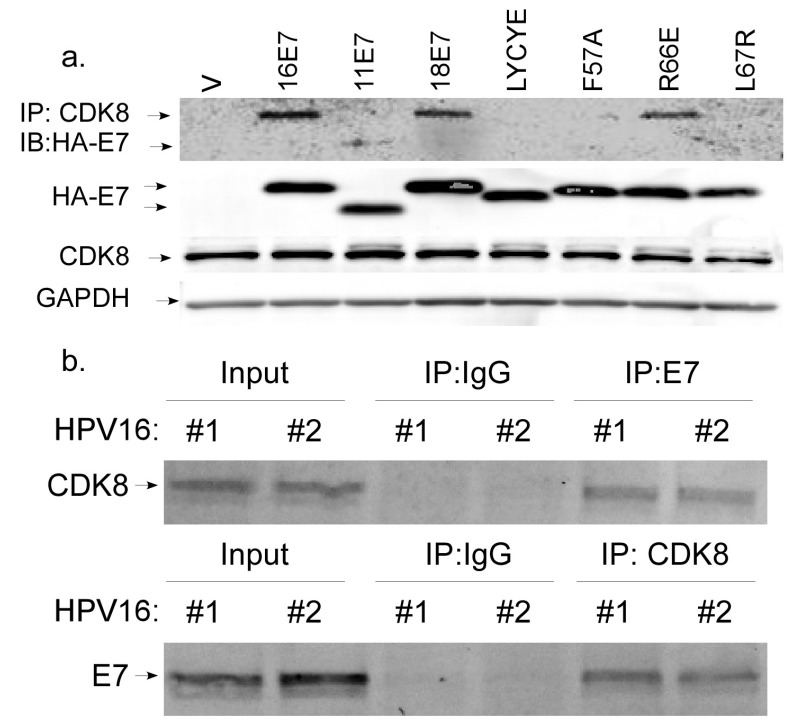
Interaction of E7 and CDK8. (**a**) Expression vectors encoding HA-tagged E7s from human papillomavirus (HPV) types 16, 11, 18, and the indicated mutants (16E7) were transfected into U2OS cells. Thirty-six hours later, total cell lysates were harvested. CDK8-containing complexes were immunoprecipitated with anti-CDK8 antibodies. Anti-HA antibody was used to detect HA-E7 by Western blotting (IB) of the immunoprecipitates (top panel). Immunoblot of total HA-E7, CDK8, and GAPDH present in the transfected U2OS cell lysates (middle panels and bottom panel) are also shown. (**b**) Immunoprecipitation was performed using total lysates from HPV16+ cells from two HFK donors with either anti-E7 antibodies (top panel), anti-CDK8 antibodies (bottom panel) or IgG (negative control). CDK8 was detected in both the anti-E7 immunoprecipitate and in the input by immunoblotting (top panel). E7 was detected in both the anti-CDK8 immunoprecipitate and in the input by immunoblotting (bottom panel).

Once nuclear, STAT1 is phosphorylated at serine 727, which is required for full induction of target genes [32,33,36]. Growing evidence suggests that Mediator kinase CDK8 is critical for IFN-induced gene expression by mediating S727 phosphorylation [31,34,67]. Previous work in our lab showed E7 and CDK8 are both critical factors in the regulation of late viral gene expression [55,68]. In follow-up studies, we investigated whether E7 associates with CDK8. U2OS cells were transfected with expression plasmids for HA-tagged HPV16E7. CDK8 was immunoprecipitated and Western blot was performed for HA-tagged HPV16E7 using anti-HA antibody. E7 was detected in the immunoprecipitate, indicating that E7 and CDK8 can associate in cells (Figure 5a). The E7s from HPV18 and HPV11 also associated with CDK8, although 11E7 associated much less efficiently. E7/CDK8 association was confirmed using lysates from keratinocytes containing episomal HPV16, from which E7-specific antibody could precipitate CDK8, and vice versa (Figure 5b). These findings indicate that endogenous E7 and endogenous CDK8 associate with each other in keratinocytes under physiological expression conditions. When expression plasmids for various E7 mutants were transfected into U2OS cells, we found that E7 F57A showed little-to-no association with CDK8 (Figure 5a) indicating that the F57 residue is required for proper association with CDK8. We found that the LYCYE deletion mutant also had reduced association with CDK8, suggesting that the N terminus of E7 participates in the association. The L67R mutation, which disrupts the overall structure of the C terminus, also disrupted association of CDK8 with E7, confirming that the C terminus is important. The R66E mutation in the CR3 domain did not disrupt E7/CDK8 association, indicating certain CR3 residues are not required for E7/CDK8 association (Figure 5a).

**Figure 6:**
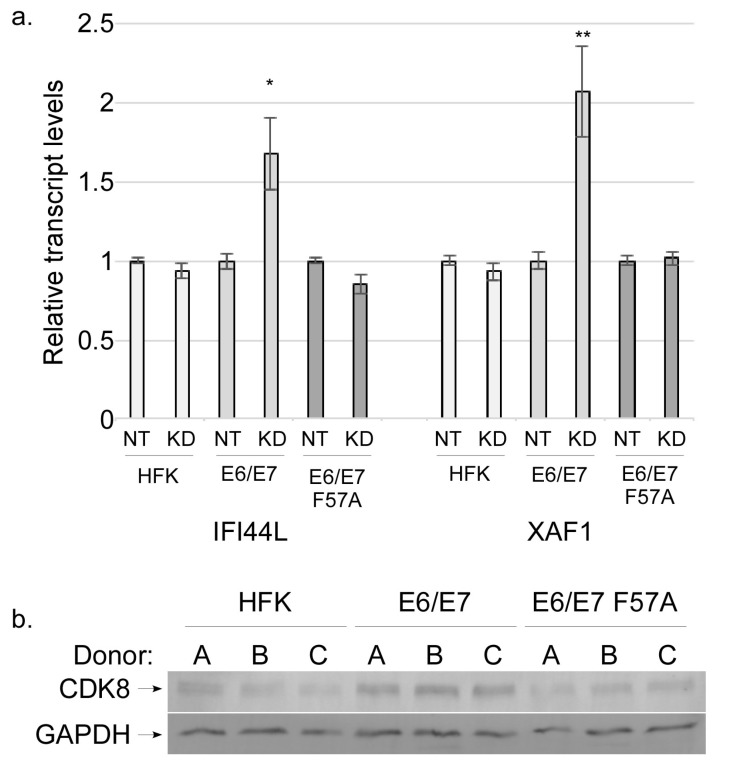
Effect of CDK8 knockdown on ISG levels. (**a**) RT-qPCR analysis of IFI44L and XAF1 transcript levels in non-target (NT) or CDK8 knockdown (KD) HFK, pLXSN E6/E7, and pLXSN E6/E7 F57A cells. Transcripts were normalized to cyclophilin A housekeeping gene with NTC values for each cell line set to 1. (**b**) Western blot analysis of CDK8 and GAPDH protein levels in HFK, pLXSN E6/E7, and pLXSN E6/E7 F57A cells from three HFK donor backgrounds.

As CDK8 regulates the expression of numerous host genes, we reasoned that interaction between E7 and CDK8 may be a novel mechanism by which E7 regulates host gene expression. Therefore, we choose to investigate whether CDK8 contributes to the ability of E7 to suppress ISG expression. To test the contribution of CDK8 to ISG expression we transfected HFK or pLXSN cell lines with siRNA to knockdown CDK8. Similar KD efficiency occurred in all cell lines (Figure S3a,b). RT-qPCR was performed to measure ISG levels upon CDK8 knockdown. ISG transcripts were significantly increased in CDK8 KD E6/E7 cells as compared to NT controls, demonstrating that CDK8 negatively regulates ISG expression in the presence of wild type E7 (Figure 6a and Figure S3c). In contrast, loss of CDK8 in HFK and pLXSN E6/E7 F57A cells did not cause an increase in ISG expression when compared to their respective NT controls (Figure 6a and Figure S3c). In support of the idea that E7/CDK8 association is important for E7-mediated regulation of host gene expression, Western blot analysis revealed significantly higher CDK8 levels in E6/E7 cells when compared to HFKs and E6/E7 F57A cells, indicating that E7 may increase CDK8 protein levels (Figure 6b). Together, these findings suggest that E7 may co-opt the transcriptional suppression ability of CDK8 to reduce the transcription of ISGs.

### 3.6. CDK8 Occupies the Promoters of Interferon-Stimulated Genes

We used the CDK8 KD samples to determine whether CDK8 mediates S727 phosphorylation in our cell lines. Western blot was performed for pS727 and showed no change in pS727 levels in HFKs, E6/E7 or E6/E7 F57A cell lines with KD of CDK8, as compared to their respective NT controls (Figure S4a,b) demonstrating that CDK8 is not the kinase responsible for S727 phosphorylation in keratinocytes under these conditions. Additionally, if E7 requires the kinase activity of CDK8 to suppress ISGs, then inhibition of CDK8 kinase activity with Senexin A (CDK8/19 kinase inhibitor) would result in increased ISGs. We found that in E6/E7 cells treated with Senexin A ISG suppression was not relieved as compared to vehicle-treated E6/E7 cells (Figure S4c). These data indicate that E7 does not require CDK8’s kinase activity to suppress ISGs.

**Figure 7:**
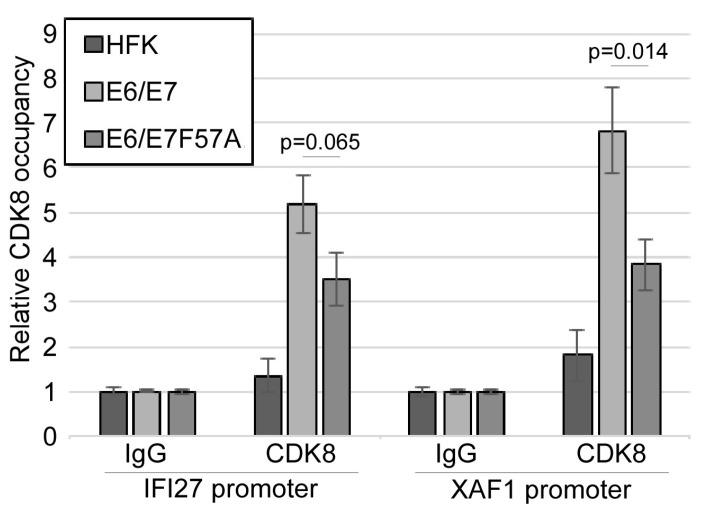
Chromatin immunoprecipitation of CDK8 from ISG promoters. ChIP was performed with IgG (control) or CDK8-specific antibodies using chromatin from HFK, pLXSN E6/E7, and pLXSN E6/E7 F57A cells. Eluted DNA from ChIP was used in a qPCR reaction with primers specific for the IFI27 and XAF1 promoters. Relative enrichment of CDK8 was determined by normalizing the CDK8 signal to IgG, with IgG samples set to 1.

CDK8 often functions in a kinase-independent manner as a component of transcriptional complexes at the promoters of target genes and during different stages of transcription, such as transcript elongation [50,69,70,71,72]. Importantly, CDK8 can either activate or inhibit gene expression depending on the specific context [50,71,72]. To begin to determine how E7 may alter CDK8 function, the ISRE region of the IFI27 and XAF1 promoters [73] in HFK and pLXSN cell lines were analyzed for CDK8 occupancy using chromatin immunoprecipitation (ChIP) coupled with quantitative PCR (qPCR). CDK8 occupies the promoters of both ISGs in the pLXSN cell lines, but CDK8 association with these promoters was not observed in HFKs (Figure 7). There was a significant loss of CDK8 enrichment at the XAF1 promoter in E6/E7 F57A cells, as compared to E6/E7 cells. Similarly, there was a reduction in CDK8 enrichment at the IFI27 promoter that approaches significance (*p* = 0.065), when compared to the CDK8 signal in E6/E7 cells. Enrichment of CDK8 at ISG promoters in E6/E7 F57A cells, as compared to HFKs may be due to residual association between E7 and CDK8. These data suggest that E7 promotes the association of CDK8 with ISG promoters, which may direct the transcriptional suppression activity of CDK8 toward these promoters. In contrast, loss of full interaction between E7 and CDK8 due to the F57A mutation disrupts the ability of E7 to promote CDK8 occupancy at these promoters, resulting in higher gene expression.

## 4. Discussion

**Figure 8:**
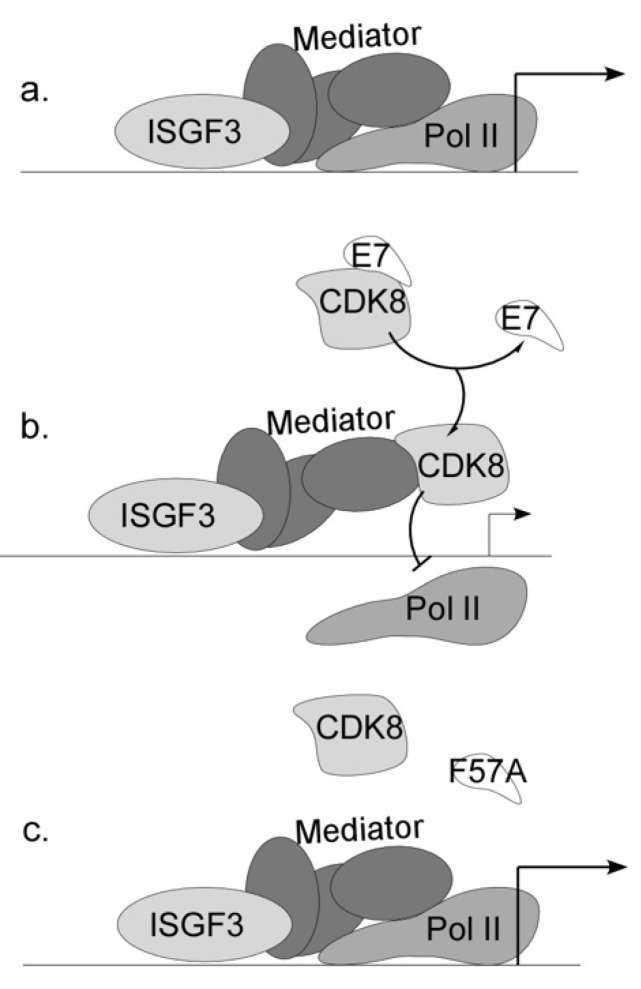
Model for the transcriptional suppression of ISGs by E7. (**a**) Factors necessary for transcription, such as Mediator and Pol II, are present at the promoters of ISGs in HFKs, and transcription occurs. (**b**) Wild type E7 associates with and recruits CDK8 to ISG promoters and this results in gene suppression, possibly due to loss of Pol II recruitment. (**c**) In cells containing F57A mutant E7, CDK8 is not efficiently recruited to ISG promoters due to reduced association between E7 F57A and CDK8. ISGs remain activated and expressed.

Our findings suggest a novel mechanism by which E7 transcriptionally suppresses a subset of IFN-stimulated genes by binding to and regulating the function of host Mediator kinase CDK8. The outcome of binding is the transcriptional suppression of a subset of ISGs. Our model proposes ISGs are constitutively expressed in keratinocytes, thus the transcriptional machinery for ISG expression is already present at ISG promoters (Figure 8). In HPV-containing cells, E7 can block expression of these activated genes through the recruitment of CDK8 to the ISG promoter. Once localized at the promoter, CDK8 is poised to suppress transcription. As E7 F57A has reduced binding capacity with CDK8, there is less CDK8 present at ISG promoters and greater gene expression.

A critical aspect of the HPV life cycle is the maintenance of the viral episome, and integration of the viral genome into host DNA is a dead end for the virus. Chronic activation of IFN signaling is one form of stress that can induce integration of the viral episome [56,62,63,64]. We found that in the presence of certain CR3 mutations, genome integration occurs at higher frequency than that of wild type HPV16 genomes (Table 1), leading us to the hypothesis that certain CR3 mutations abrogate E7’s ability to suppress immune-related genes. To test this, we performed transcriptome analysis of cells containing the E7 F57A mutant compared to cells with wild type E7. We found that certain ISGs were more highly expressed in cells containing the E7 F57A mutant as compared to cells containing wild type E7 (Table 3 and Figure 2). Notably, it was a small subset of ISGs as opposed to the potentially hundreds of ISGs that can be induced by type I IFN signaling. ISGs have a variety of anti-viral activities, some of which have negative consequences for host cell survival, such as by promoting apoptosis [74]. As keratinocytes have a basal level of ISG expression in the absence of infection, we speculate that the ISGs chronically expressed by HFKs (and thus targeted by E7) must be ones that are well-tolerated by keratinocytes long-term. Future studies are needed to know whether E7 specifically targets these ISGs or suppresses all activated ISGs in HFKs.

As we measured robust ISG levels in HFK and E6/E7 F57A cells, and these genes are typically induced in response to the JAK/STAT pathway downstream of type I IFN signaling, we expected to find differences in STAT1 activation when compared to E6/E7 cells. Instead we found that Y701 phosphorylation was usually absent in keratinocytes (Figure 4a). These data support the findings of others that expression of ISGs is possible despite the absence of STAT1 tyrosine phosphorylation [66,73,75]. Studies from the Stark lab have shown that chronic IFN signaling increases levels of unphosphorylated STATs (U-STAT1 and U-STAT2) that form an unphosphorylated ISGF3 complex in conjunction with IRF9 (U-ISGF3) that activates transcription of a subset of the ISGs [66,73]. We reason that the constitutive production of IFN-κ by keratinocytes may induce U-ISGF3 complexes, and investigation into this possibility may serve as the basis of future studies. While pS727 levels were seemingly higher in HFK and E6/E7 F57A cells than E6/E7 cells, analysis of total STAT1 followed a similar trend, leading us to the interpretation that E7 does not regulate STAT1 activation to suppress ISG expression, but rather that STAT1 transcript levels are regulated similarly to those of other ISGs (Figure 4). STAT1 S727 phosphorylation occurs following re-localization of the ISGF3 complex to the nucleus [33]. As pS727 was detected in cells containing E7, the ability of E7 to interact with IRF9 to prevent nuclear localization [39] may not play a major role in IFN suppression in these cell lines, though more studies are needed to determine whether E7 F57A retains the ability to interact with IRF9. Despite the absence of pY701 STAT1 in these cells, ISGs (except IFI6) required JAK activity for full expression, as predicted by canonical IFN signaling (Figure 3 and Figure S2). More research is required to understand the importance of JAK activity to ISG induction in the absence of pY701. A larger point, however, is that ISGs, which are often thought of as a homogenously regulated block of genes, can actually be subject to different regulatory controls. The significance of these differences in regulation are poorly understood at best. These data highlight that the regulation of IFN signaling is poorly understood in keratinocytes and suggest there might be unique regulatory mechanisms that exist for certain ISG subsets. 

Different candidate kinases have been proposed to mediate S727 phosphorylation. In response to various cellular stresses, p38 mitogen-activated protein kinase (MAPK) phosphorylates S727 [76]. Protein kinase C delta (PCK-δ) was reported to mediate phosphorylation of S727 in response to type I IFN, however its contribution to S727 phosphorylation in vivo has not been researched [35]. Mediator kinase CDK8 is required for the expression of genes induced by IFN-γ stimulation through the phosphorylation of S727 [31,34,67]. We found that in keratinocytes CDK8 does not contribute to STAT1 activation through S727 phosphorylation (Figure S4a,b). It was also notable that S727 phosphorylation was readily detectible, even in the absence of pY701 (Figure 4). Determining which kinase mediates S727 phosphorylation in keratinocytes will require additional studies.

CDK8 is well known to have dual regulatory roles as an activator or repressor of transcription, depending on promoter context [71,72]. These activities can either be independent or dependent on its kinase activity [71,72,77,78]. For example, CDK8 is a co-activator of the p21 gene and is recruited to the promoter upon p53 activation [52]. CDK8 directly activates specific transcription factors through phosphorylation, such as STAT1 [31]. CDK8-mediated phosphorylation of Smad transcription factors activates their transcriptional activity but concomitantly increases protein turnover [79]. For other transcription factors, such as SREBP-1c (sterol regulatory element-binding protein 1c), phosphorylation by CDK8 only enhances protein turn over, thus making CDK8 a negative regulator [80]. At the promoter, CDK8 can also repress transcription through association with Mediator, as binding of CDK8 to Mediator causes a structural shift in Mediator that prevents the ability of Pol II to associate with the complex [51]. Beyond the promoter, CDK8 positively regulates the expression of HIF1α-regulated genes and serum response genes by promoting productive transcriptional elongation [53,70]. These various activities of CDK8 make it difficult to predict ahead of time what effect it could have on a given gene.

We found that E7 requires CDK8 to suppress ISGs, so that knockdown of CDK8 in E6/E7 cells resulted in increased ISG transcripts, when compared to NT controls (Figure 6 and Figure S3). Suppression of transcription was correlated with recruitment of CDK8 to promoters, and this recruitment was disrupted by the F57A mutation (Figure 7). The nature of CDK8’s suppressive mechanism is not yet clear. Our data suggest that CDK8 kinase activity does not contribute to ISG suppression by E7 (Figure S4c), which indicates phosphorylation of transcriptional regulators by CDK8 is not a point of ISG regulation by E7. The CDK8 submodule through its MED12 subunit associates with G9a, a histone methyltransferase, to direct H3K9 methylation to suppress transcription [54]. However, preliminary data indicate that methylation of ISG promoters is not different between E6/E7 and E6/E7 F57A cell lines. It is possible that the presence of CDK8 simply prevents the proper association of Pol II with the Mediator complex [51]. Future studies are required to validate this model and should include further characterization of which factors occupy ISG promoters, the molecular mechanism of CDK8 recruitment to ISG promoters by E7, and investigation into how CDK8 occupancy contributes to transcriptional suppression.

The ability to interact with host proteins is critical to E7’s biological functions due to the lack of intrinsic enzymatic activity of E7 [14]. E7 often functions by recruiting or displacing transcriptional regulators to alter host gene expression [16]. We are the first to show that CDK8 is an interacting partner of E7 (Figure 5), adding to the repertoire of interacting partners that are transcriptional regulators. E7 may interact with other components of Mediator, possibly indirectly through CDK8. Supporting this idea, E1A encoded by Adenovirus shows homology to E7 [14] and has been shown to associate with subunits of Mediator [81,82,83,84], enhancing the transcription potential of E1A [83,84]. We speculate that CDK8 is an advantageous interacting partner due to the ability of CDK8 to reversibly associate with Mediator, which in part contributes to CDK8’s dual functionality as a transcriptional activator and repressor. Perhaps E7 can exploit this feature by making CDK8 associate or disassociate as needed in response to various cellular conditions to favor gene expression patterns that support the viral life cycle.

## Figures and Tables

**Table 1:** Tabulation of Southern blots showing episomal maintenance of viral DNA in wild type HPV16+ or CR3 mutant cells.

Genotype	Number of Samples	Episomal	Integrated	Fraction Episomal	*p* Value (Wt v Mutant)
HPV16 (wild type)	51	40	11	0.78	
M84S	8	4	4	0.50	0.18
G85A	6	3	3	0.50	0.15
Y52A	10	5	5	0.50	0.11
ED80-81KK	5	2	3	0.40	0.09
D62K	7	3	4	0.43	0.07
N53D	12	6	6	0.50	0.07
S63D	9	3	6	0.33	<0.05
QKP96-98EEA	11	3	8	0.27	<0.01
V55T	13	4	9	0.31	<0.01
T64D	7	1	6	0.14	<0.01
R77E	7	0	7	0.00	<0.001
R66E	13	2	11	0.15	<0.0001
F57A	34	6	28	0.18	<0.0001

**Table 2:** Enriched pathways based on upregulated genes in E6/E7 F57A cells (compared to pLXSN E6/E7 cells).

Pathway Name	# Entities Found	# Entities Total	Entities FDR
Interferon alpha/beta signaling	10	184	<0.0001
Interferon Signaling	10	392	<0.0001
Cytokine Signaling in Immune system	11	1055	<0.001
Transport of fatty acids	2	18	0.01
Transport of vitamins, nucleosides, and related molecules	3	100	0.02

**Table 3:** RNA-sequencing results showing top 20 genes up- or downregulated in pLXSN E6/E7 F57A cells, as compared to pLXSN E6/E7.

Upregulated		Downregulated	
Gene ID	Fold Change	Gene ID	Fold Change
SLC44A5	5713.3	ARL2-SNX15	−4881.7
GPAT2	3410.2	AC013489.1	−4599.3
SLC27A6	170.4	AC139530.2	−4185.4
VAV1	147.7	UGT1A3	−4061.3
**IFI27**	49.3	AC097658.1	−441.7
KRBOX1	47.7	USP32P2	−102.9
**IFI44L**	34.3	MTND1P23	−69.8
**XAF1**	29.8	SHC1P1	−59.0
**IFI6**	23.7	FOXF2	-49.8
**MX2**	22.6	MAMLD1	−43.6
FYB1	19.5	FAM103A2P	−30.7
FAM26E	19.1	SPINK7	−30.1
**BST2**	17.7	RAB4B-EGLN2	−30.0
AL163051.1	10.6	SPRR4	−28.9
AC009268.2	8.1	AC008556.1	−26.6
AL136295.5	7.6	VASH2	−21.7
AL450992.2	7.4	SORD2P	−18.7
PART1	3.4	LYPD2	−17.7
AC010326.4	3.1	SEPT7P7	−17.2
AC022400.7	2.5	PKNOX2	−14.2

## Supplementary Materials

The following are available online at https://www.mdpi.com/1999-4915/12/3/311/s1, Figure S1: Growth rate and integration of CR3 mutants, Figure S2: Effect of Ruxolitinib on ISG levels, Figure S3: Effect of CDK8 knockdown on ISG expression, Figure S4: Effect of CDK8 kinase inhibition on ISG expression, Table S1: Oligos used in this study, File S1: Unfiltered RNA-sequencing results of genes differentially expressed in pLXSN E6/E7 F57A cells, as compared to pLXSN E6/E7 cells, File S2: Reactome analysis of RNA-sequencing results of genes differentially expressed in pLXSN E6/E7 F57A cells, as compared to pLXSN E6/E7 cells.

## References

[1] Moscicki A.B., Schiffman M., Burchell A., Albero G., Giuliano A.R., Goodman M.T., Kjaer S.K., Palefsky J. (2012). Updating the natural history of human papillomavirus and anogenital cancers. Vaccine.

[2] Doorbar J., Egawa N., Griffin H., Kranjec C., Murakami I. (2015). Human papillomavirus molecular biology and disease association. Rev. Med. Virol..

[3] Clifford G.M., Smith J.S., Aguado T., Franceschi S. (2003). Comparison of HPV type distribution in high-grade cervical lesions and cervical cancer: A meta-analysis. Br. J. Cancer.

[4] McBride A.A. (2017). Mechanisms and strategies of papillomavirus replication. Biol. Chem..

[5] Longworth M.S., Laimins L.A. (2004). Pathogenesis of human papillomaviruses in differentiating epithelia. Microbiol. Mol. Biol. Rev..

[6] Moody C. (2017). Mechanisms by which HPV Induces a Replication Competent Environment in Differentiating Keratinocytes. Viruses.

[7] Bodily J., Laimins L.A. (2011). Persistence of human papillomavirus infection: Keys to malignant progression. Trends Microbiol..

[8] Gielen V., Schmitt D., Thivolet J. (1988). HLA class I antigen (heavy and light chain) expression by Langerhans cells and keratinocytes of the normal human epidermis: Ultrastructural quantitation using immunogold labelling procedure. Arch. Dermatol. Res..

[9] Kubo A., Nagao K., Yokouchi M., Sasaki H., Amagai M. (2009). External antigen uptake by Langerhans cells with reorganization of epidermal tight junction barriers. J. Exp. Med..

[10] Woodby B.L., Songock W.K., Scott M.L., Raikhy G., Bodily J.M. (2018). Induction of Interferon Kappa in Human Papillomavirus 16 Infection by Transforming Growth Factor Beta-Induced Promoter Demethylation. J. Virol..

[11] Halbert C.L., Demers G.W., Galloway D.A. (1991). The E7 gene of human papillomavirus type 16 is sufficient for immortalization of human epithelial cells. J. Virol..

[12] Munger K., Basile J.R., Duensing S., Eichten A., Gonzalez S.L., Grace M., Zacny V.L. (2001). Biological activities and molecular targets of the human papillomavirus E7 oncoprotein. Oncogene.

[13] Vousden K.H., Vojtesek B., Fisher C., Lane D. (1993). HPV-16 E7 or adenovirus E1A can overcome the growth arrest of cells immortalized with a temperature-sensitive p53. Oncogene.

[14] Roman A., Munger K. (2013). The papillomavirus E7 proteins. Virology.

[15] Westrich J.A., Warren C.J., Pyeon D. (2017). Evasion of host immune defenses by human papillomavirus. Virus. Res..

[16] Songock W.K., Kim S.M., Bodily J.M. (2017). The human papillomavirus E7 oncoprotein as a regulator of transcription. Virus. Res..

[17] Bodily J.M., Mehta K.P., Laimins L.A. (2011). Human papillomavirus E7 enhances hypoxia-inducible factor 1-mediated transcription by inhibiting binding of histone deacetylases. Cancer Res..

[18] Bernat A., Avvakumov N., Mymryk J.S., Banks L. (2003). Interaction between the HPV E7 oncoprotein and the transcriptional coactivator p300. Oncogene.

[19] Brehm A., Nielsen S.J., Miska E.A., McCance D.J., Reid J.L., Bannister A.J., Kouzarides T. (1999). The E7 oncoprotein associates with Mi2 and histone deacetylase activity to promote cell growth. EMBO J..

[20] Burgers W.A., Blanchon L., Pradhan S., de Launoit Y., Kouzarides T., Fuks F. (2007). Viral oncoproteins target the DNA methyltransferases. Oncogene.

[21] Massimi P., Pim D., Banks L. (1997). Human papillomavirus type 16 E7 binds to the conserved carboxy-terminal region of the TATA box binding protein and this contributes to E7 transforming activity. J. Gen. Virol..

[22] Park J.S., Kim E.J., Kwon H.J., Hwang E.S., Namkoong S.E., Um S.J. (2000). Inactivation of interferon regulatory factor-1 tumor suppressor protein by HPV E7 oncoprotein. Implication for the E7-mediated immune evasion mechanism in cervical carcinogenesis. J. Biol. Chem..

[23] McLaughlin-Drubin M.E., Huh K.W., Munger K. (2008). Human papillomavirus type 16 E7 oncoprotein associates with E2F6. J. Virol..

[24] Boyer S.N., Wazer D.E., Band V. (1996). E7 protein of human papilloma virus-16 induces degradation of retinoblastoma protein through the ubiquitin-proteasome pathway. Cancer Res..

[25] Chellappan S., Kraus V.B., Kroger B., Munger K., Howley P.M., Phelps W.C., Nevins J.R. (1992). Adenovirus E1A, simian virus 40 tumor antigen, and human papillomavirus E7 protein share the capacity to disrupt the interaction between transcription factor E2F and the retinoblastoma gene product. Proc. Natl. Acad. Sci. USA.

[26] Munger K., Werness B.A., Dyson N., Phelps W.C., Harlow E., Howley P.M. (1989). Complex formation of human papillomavirus E7 proteins with the retinoblastoma tumor suppressor gene product. EMBO J..

[27] LaFleur D.W., Nardelli B., Tsareva T., Mather D., Feng P., Semenuk M., Taylor K., Buergin M., Chinchilla D., Roshke V. (2001). Interferon-kappa, a novel type I interferon expressed in human keratinocytes. J. Biol. Chem..

[28] Ivashkiv L.B., Donlin L.T. (2014). Regulation of type I interferon responses. Nat. Rev. Immunol..

[29] Qureshi S.A., Salditt-Georgieff M., Darnell J.E. (1995). Tyrosine-phosphorylated Stat1 and Stat2 plus a 48-kDa protein all contact DNA in forming interferon-stimulated-gene factor 3. Proc. Natl. Acad. Sci. USA.

[30] Wang W., Xu L., Su J., Peppelenbosch M.P., Pan Q. (2017). Transcriptional Regulation of Antiviral Interferon-Stimulated Genes. Trends Microbiol..

[31] Bancerek J., Poss Z.C., Steinparzer I., Sedlyarov V., Pfaffenwimmer T., Mikulic I., Dolken L., Strobl B., Muller M., Taatjes D.J. (2013). CDK8 kinase phosphorylates transcription factor STAT1 to selectively regulate the interferon response. Immunity.

[32] Pilz A., Ramsauer K., Heidari H., Leitges M., Kovarik P., Decker T. (2003). Phosphorylation of the Stat1 transactivating domain is required for the response to type I interferons. EMBO Rep..

[33] Sadzak I., Schiff M., Gattermeier I., Glinitzer R., Sauer I., Saalmuller A., Yang E., Schaljo B., Kovarik P. (2008). Recruitment of Stat1 to chromatin is required for interferon-induced serine phosphorylation of Stat1 transactivation domain. Proc. Natl. Acad. Sci. USA.

[34] Steinparzer I., Sedlyarov V., Rubin J.D., Eislmayr K., Galbraith M.D., Levandowski C.B., Vcelkova T., Sneezum L., Wascher F., Amman F. (2019). Transcriptional Responses to IFN-gamma Require Mediator Kinase-Dependent Pause Release and Mechanistically Distinct CDK8 and CDK19 Functions. Mol. Cell.

[35] Uddin S., Sassano A., Deb D.K., Verma A., Majchrzak B., Rahman A., Malik A.B., Fish E.N., Platanias L.C. (2002). Protein kinase C-delta (PKC-delta ) is activated by type I interferons and mediates phosphorylation of Stat1 on serine 727. J. Biol. Chem..

[36] Wen Z., Zhong Z., Darnell J.E. (1995). Maximal activation of transcription by Stat1 and Stat3 requires both tyrosine and serine phosphorylation. Cell.

[37] Schneider W.M., Chevillotte M.D., Rice C.M. (2014). Interferon-stimulated genes: A complex web of host defenses. Annu. Rev. Immunol..

[38] Antonsson A., Payne E., Hengst K., McMillan N.A. (2006). The human papillomavirus type 16 E7 protein binds human interferon regulatory factor-9 via a novel PEST domain required for transformation. J. Interferon Cytokine Res..

[39] Barnard P., McMillan N.A. (1999). The human papillomavirus E7 oncoprotein abrogates signaling mediated by interferon-alpha. Virology.

[40] Hong S., Mehta K.P., Laimins L.A. (2011). Suppression of STAT-1 expression by human papillomaviruses is necessary for differentiation-dependent genome amplification and plasmid maintenance. J. Virol..

[41] Nees M., Geoghegan J.M., Hyman T., Frank S., Miller L., Woodworth C.D. (2001). Papillomavirus type 16 oncogenes downregulate expression of interferon-responsive genes and upregulate proliferation-associated and NF-kappaB-responsive genes in cervical keratinocytes. J. Virol..

[42] Perea S., Lopezocejo O., Vongabain A., Arana M. (1997). Human papillomavirus type-16 (HPV-16) major transforming proteins functionally interact with interferon signaling mechanisms. Int. J. Oncol..

[43] Shaikh M.H., Bortnik V., McMillan N.A., Idris A. (2019). cGAS-STING responses are dampened in high-risk HPV type 16 positive head and neck squamous cell carcinoma cells. Microb. Pathog..

[44] Um S.J., Rhyu J.W., Kim E.J., Jeon K.C., Hwang E.S., Park J.S. (2002). Abrogation of IRF-1 response by high-risk HPV E7 protein in vivo. Cancer Lett..

[45] Bodily J.M., Mehta K.P., Cruz L., Meyers C., Laimins L.A. (2011). The E7 open reading frame acts in cis and in trans to mediate differentiation-dependent activities in the human papillomavirus type 16 life cycle. J. Virol..

[46] Edmonds C., Vousden K.H. (1989). A point mutational analysis of human papillomavirus type 16 E7 protein. J. Virol..

[47] Todorovic B., Massimi P., Hung K., Shaw G.S., Banks L., Mymryk J.S. (2011). Systematic analysis of the amino acid residues of human papillomavirus type 16 E7 conserved region 3 involved in dimerization and transformation. J. Virol..

[48] Fant C.B., Taatjes D.J. (2019). Regulatory functions of the Mediator kinases CDK8 and CDK19. Transcription.

[49] Meyer K.D., Donner A.J., Knuesel M.T., York A.G., Espinosa J.M., Taatjes D.J. (2008). Cooperative activity of cdk8 and GCN5L within Mediator directs tandem phosphoacetylation of histone H3. EMBO J..

[50] Nemet J., Jelicic B., Rubelj I., Sopta M. (2014). The two faces of Cdk8, a positive/negative regulator of transcription. Biochimie.

[51] Knuesel M.T., Meyer K.D., Bernecky C., Taatjes D.J. (2009). The human CDK8 subcomplex is a molecular switch that controls Mediator coactivator function. Genes Dev..

[52] Donner A.J., Szostek S., Hoover J.M., Espinosa J.M. (2007). CDK8 is a stimulus-specific positive coregulator of p53 target genes. Mol. Cell.

[53] Galbraith M.D., Allen M.A., Bensard C.L., Wang X., Schwinn M.K., Qin B., Long H.W., Daniels D.L., Hahn W.C., Dowell R.D. (2013). HIF1A employs CDK8-mediator to stimulate RNAPII elongation in response to hypoxia. Cell.

[54] Ding N., Zhou H., Esteve P.O., Chin H.G., Kim S., Xu X., Joseph S.M., Friez M.J., Schwartz C.E., Pradhan S. (2008). Mediator links epigenetic silencing of neuronal gene expression with x-linked mental retardation. Mol. Cell.

[55] Bodily J.M., Hennigan C., Wrobel G.A., Rodriguez C.M. (2013). Regulation of the human papillomavirus type 16 late promoter by E7 and the cell cycle. Virology.

[56] Scott M.L., Woodby B.L., Ulicny J., Raikhy G., Orr A.W., Songock W.K., Bodily J.M. (2019). Human papillomavirus type 16 E5 inhibits interferon signaling and supports episomal viral maintenance. J. Virol..

[57] Wilson R., Laimins L.A. (2005). Differentiation of HPV-containing cells using organotypic "raft" culture or methylcellulose. Methods Mol. Med..

[58] Fabregat A., Jupe S., Matthews L., Sidiropoulos K., Gillespie M., Garapati P., Haw R., Jassal B., Korninger F., May B. (2018). The Reactome Pathway Knowledgebase. Nucleic Acids Res..

[59] Fabregat A., Sidiropoulos K., Viteri G., Forner O., Marin-Garcia P., Arnau V., D’Eustachio P., Stein L., Hermjakob H. (2017). Reactome pathway analysis: A high-performance in-memory approach. BMC Bioinform..

[60] McIntyre M.C., Frattini M.G., Grossman S.R., Laimins L.A. (1993). Human papillomavirus type 18 E7 protein requires intact Cys-X-X-Cys motifs for zinc binding, dimerization, and transformation but not for Rb binding. J. Virol..

[61] Liu X., Clements A., Zhao K., Marmorstein R. (2006). Structure of the human Papillomavirus E7 oncoprotein and its mechanism for inactivation of the retinoblastoma tumor suppressor. J. Biol. Chem..

[62] Chang Y.E., Pena L., Sen G.C., Park J.K., Laimins L.A. (2002). Long-term effect of interferon on keratinocytes that maintain human papillomavirus type 31. J. Virol..

[63] Herdman M.T., Pett M.R., Roberts I., Alazawi W.O., Teschendorff A.E., Zhang X.Y., Stanley M.A., Coleman N. (2006). Interferon-beta treatment of cervical keratinocytes naturally infected with human papillomavirus 16 episomes promotes rapid reduction in episome numbers and emergence of latent integrants. Carcinogenesis.

[64] Pett M., Coleman N. (2007). Integration of high-risk human papillomavirus: A key event in cervical carcinogenesis?. J. Pathol..

[65] Bordignon V., Di Domenico E.G., Trento E., D’Agosto G., Cavallo I., Pontone M., Pimpinelli F., Mariani L., Ensoli F. (2017). How Human Papillomavirus Replication and Immune Evasion Strategies Take Advantage of the Host DNA Damage Repair Machinery. Viruses.

[66] Cheon H., Stark G.R. (2009). Unphosphorylated STAT1 prolongs the expression of interferon-induced immune regulatory genes. Proc. Natl. Acad. Sci. USA.

[67] Staab J., Herrmann-Lingen C., Meyer T. (2013). CDK8 as the STAT1 serine 727 kinase?. JAK-STAT.

[68] Songock W.K., Scott M.L., Bodily J.M. (2017). Regulation of the human papillomavirus type 16 late promoter by transcriptional elongation. Virology.

[69] Akoulitchev S., Chuikov S., Reinberg D. (2000). TFIIH is negatively regulated by cdk8-containing mediator complexes. Nature.

[70] Donner A.J., Ebmeier C.C., Taatjes D.J., Espinosa J.M. (2010). CDK8 is a positive regulator of transcriptional elongation within the serum response network. Nat. Struct. Mol. Biol..

[71] Galbraith M.D., Donner A.J., Espinosa J.M. (2010). CDK8: A positive regulator of transcription. Transcription.

[72] Taatjes D.J. (2010). The human Mediator complex: A versatile, genome-wide regulator of transcription. Trends Biochem. Sci..

[73] Cheon H., Holvey-Bates E.G., Schoggins J.W., Forster S., Hertzog P., Imanaka N., Rice C.M., Jackson M.W., Junk D.J., Stark G.R. (2013). IFNbeta-dependent increases in STAT1, STAT2, and IRF9 mediate resistance to viruses and DNA damage. EMBO J..

[74] Borden E.C., Sen G.C., Uze G., Silverman R.H., Ransohoff R.M., Foster G.R., Stark G.R. (2007). Interferons at age 50: Past, current and future impact on biomedicine. Nat. Rev. Drug Discov..

[75] Sung P.S., Cheon H., Cho C.H., Hong S.H., Park D.Y., Seo H.I., Park S.H., Yoon S.K., Stark G.R., Shin E.C. (2015). Roles of unphosphorylated ISGF3 in HCV infection and interferon responsiveness. Proc. Natl. Acad. Sci. USA.

[76] Kovarik P., Stoiber D., Eyers P.A., Menghini R., Neininger A., Gaestel M., Cohen P., Decker T. (1999). Stress-induced phosphorylation of STAT1 at Ser727 requires p38 mitogen-activated protein kinase whereas IFN-gamma uses a different signaling pathway. Proc. Natl. Acad. Sci. USA.

[77] Allen B.L., Taatjes D.J. (2015). The Mediator complex: A central integrator of transcription. Nat. Rev. Mol. Cell Biol..

[78] Poss Z.C., Ebmeier C.C., Odell A.T., Tangpeerachaikul A., Lee T., Pelish H.E., Shair M.D., Dowell R.D., Old W.M., Taatjes D.J. (2016). Identification of Mediator Kinase Substrates in Human Cells using Cortistatin A and Quantitative Phosphoproteomics. Cell Rep..

[79] Alarcon C., Zaromytidou A.I., Xi Q., Gao S., Yu J., Fujisawa S., Barlas A., Miller A.N., Manova-Todorova K., Macias M.J. (2009). Nuclear CDKs drive Smad transcriptional activation and turnover in BMP and TGF-beta pathways. Cell.

[80] Zhao X., Feng D., Wang Q., Abdulla A., Xie X.J., Zhou J., Sun Y., Yang E.S., Liu L.P., Vaitheesvaran B. (2012). Regulation of lipogenesis by cyclin-dependent kinase 8-mediated control of SREBP-1. J. Clin. Investg..

[81] Boyer T.G., Martin M.E., Lees E., Ricciardi R.P., Berk A.J. (1999). Mammalian Srb/Mediator complex is targeted by adenovirus E1A protein. Nature.

[82] Stevens J.L., Cantin G.T., Wang G., Shevchenko A., Shevchenko A., Berk A.J. (2002). Transcription control by E1A and MAP kinase pathway via Sur2 mediator subunit. Science.

[83] Vijayalingam S., Chinnadurai G. (2013). Adenovirus L-E1A activates transcription through mediator complex-dependent recruitment of the super elongation complex. J. Virol..

[84] Wang G., Berk A.J. (2002). In vivo association of adenovirus large E1A protein with the human mediator complex in adenovirus-infected and -transformed cells. J. Virol..

