# Supplementary material for: Suppression of a Subset of Interferon-Induced Genes by Human Papillomavirus Type 16 E7 via a Cyclin Dependent Kinase 8-Dependent Mechanism
Source: Viruses. 2020 Mar 13;12(3):311. doi: 10.3390/v12030311 (PMC7150855; doi:10.3390/v12030311)
Supplement: Supplementary file 1 [file viruses-12-00311-s001.zip › Supp Figures and Oligos - revised.docx]

Table S1: Oligos used in this study

| Primer | Sequence (5’-3’) |
| --- | --- |
| Cyclophilin 5’ | CTTGGGCCGC GTCTCC |
| Cyclophilin 3’ | GCAGGAACCCTTATAACCAAATCC |
| IFIT1 (p56) 5’ | TGCTCCAGACTATCCTTGACCT |
| IFIT1 (p56) 3’ | TCTCAGAGGAGCCTGGCTAA |
| IFI27 5’ | GGCAGCCTTGTGGCTACTCT |
| IFI27 3’ | ATGGAGCCCAGGATGAACTTG |
| IFI44L 5’ | GGCCATGTGACTGGCCAA |
| IFI44L 3’ | GCCCCATCTAGCCCCATAGT |
| IFI6 5’ | GTTCTCACTATATTGTCCAGGCTAGAGT |
| IFI6 3’ | AGTTTATTCTGTTGTCACATCTAGGTTGTT |
| MX2 5’ | AAACTGTTCAGAGCACGATTGAAG |
| MX2 3’ | ACCATCTGCTCCATTCTGAACTG |
| XAF1 5’ | AAGGGGAAAGAATTTCAGCTCCT |
| XAF1 3’ | CTGGATTTCCAACAGGAAAGTGTT |
| BST2 5’ | GGTGGCCCGTAGAAGATTCC |
| BST2 3’ | CTTACAGCGCTTATCCCCGT |
| Total STAT1 5’ | CCAGGCTCTTGATTTCATGC |
| Total STAT1 3’ | AATTCTGGAAAACGCCCAG |
| IFI27 promoter (CDK8 ChIP) 5’ | CTTCTGGACTGCGCATGAGG |
| IFI27 promoter (CDK8 ChIP) 3’ | CCACCCCGACTGAAGCACTG |
| XAF1 promoter (CDK8 ChIP) 5’ | CTCCTCCCTCCCTGAAGCT |
| XAF1 promoter (CDK8 ChIP) 3’ | CGGTTGAGTTTCGTTTCTTGCA |
| ED80-1KK 5' | GTAGACATTCGTACTTTGAAAAAGCTGTTAATGGGCACACTAGGAATTG |
| ED80-1KK 3' | CAATTCCTAGTGTGCCCATTAACAGCTTTTTCAAAGTACGAATGTCTAC |
| G85A 5' | GTACTTTGGAAGACCTGTTAATGGCCACACTAGGAATTGTGTGCC |
| G85A 3' | GGCACACAATTCCTAGTGTGGCCATTAACAGGTCTTCCAAAGTAC |
| Y52A 5' | CAGAACCGGACAGAGCCCATGCCAATATTGTAACCTTTTGTTGC |
| Y52A 3' | GCAACAAAAGGTTACAATATTGGCATGGGCTCTGTCCGGTTCTG |
| S63D 5' | CTTTTGTTGCAAGTGTGACGATACGCTTCGGTTGTGCGTACA |
| S63D 3' | TGTACGCACAACCGAAGCGTATCGTCACACTTGCAACAAAAG |
| N53D 5' | CAGAACCGGACAGAGCCCATTACGATATTGTAACCTTTTGTTGC |
| N53D 3' | GCAACAAAAGGTTACAATATCGTAATGGGCTCTGTCCGGTTCTG |
| QKP96-98EEA 5' | GTGCCCCATC TGTTCTGAGG AAGCATAATC TACCATGGCT |
| QKP96-98EEA 3' | AGCCATGGTAGATTATGCTTCCTCAGAACAGATGGGGCAC |
| R66E 5' | GTGTGACTCT ACGCTTGAGT TGTGCGTACA |
| R66E 3' | TGTACGCACAACTCAAGCGTAGAGTCACAC |
| R77E 5' | GTAGACATTGAG ACTTTGGA AGACCTGTTA |
| R77E 3' | TAACAGGTCTTCCAAAGTCTCAATGTCTAC |
| T64D 5' | GTGTGACTCT GACCTTCGGT TGTGCGTACA |
| T64D 3' | TGTACGCACAACCGAAGGTCAGAGTCACAC |
| V55T 5' | CCATTACAAT ATTACAACCT TTTGTTGCAA |
| V55T 3' | TTGCAACAAAAGGTTGTAATATTGTAATGG |
| F57A 5' | CCATTACAAT ATTGTAACC GCT TGTTGCAA GTGTGACTCT ACGCT |
| F57A 3' | AGCGTAGAGTCACACTTGCAACAAGCGGTTACAATATTGTAATGG |
| D62K 5' | TTTGTTGCAA GTGTAAGTCT ACGCTTCGGT |
| D62K 3' | ACCGAAGCGTAGACTTACACTTGCAACAAA |
| M84S 5' | AGACCTGTTA AGCGGCACAC TAGGAATTGT |
| M84S 3' | ACAATTCCTAGTGTGCCGCTTAACAGGTCT |
| 16E7 Xho frame 5’ | cgcctcgagcatggagatacacctaca |
| 16E7 Not stop 3’ | gcg gcggccgcttatggtttctgagaaca |


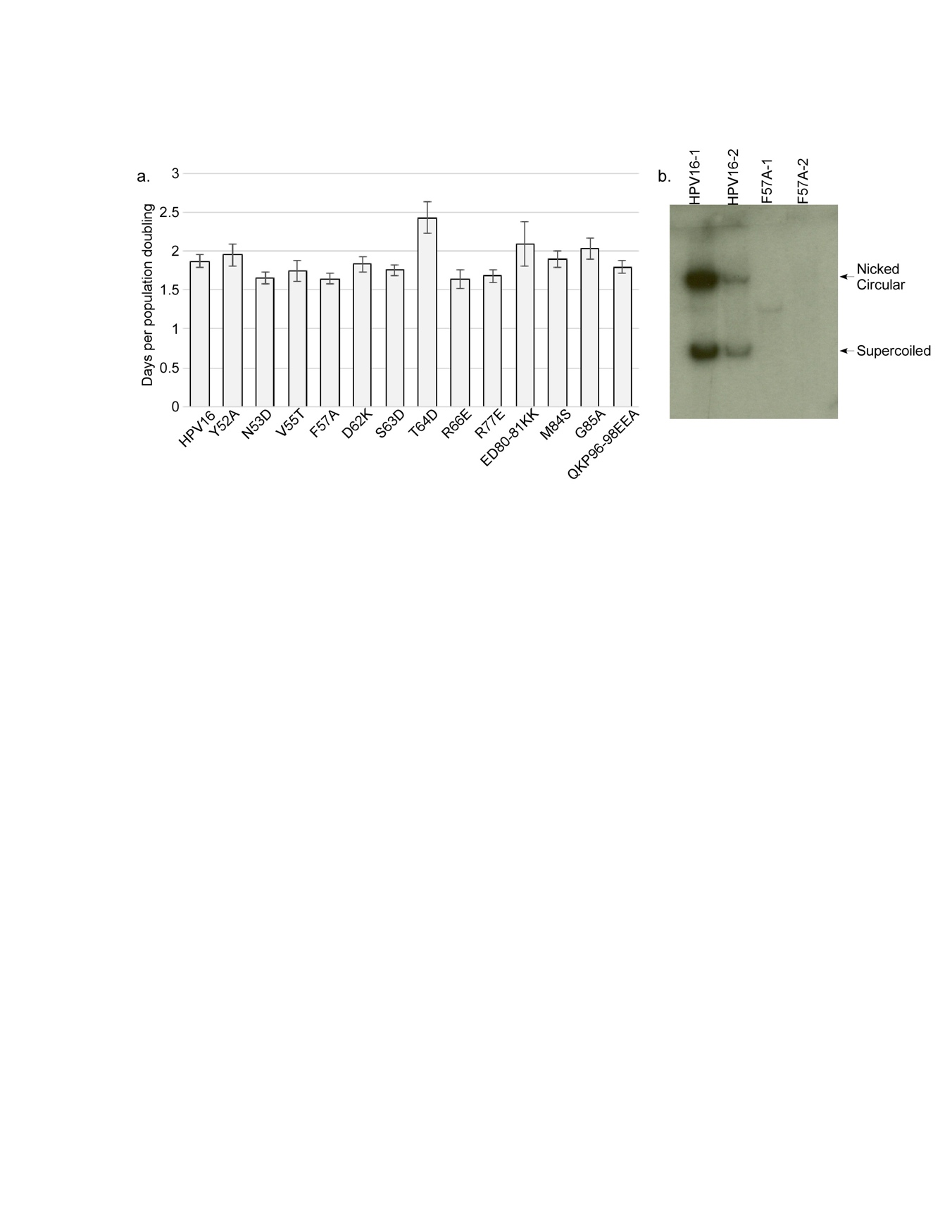
Figure S1: Growth rate and integration of CR3 mutants. (a) Growth rate of human foreskin keratinocytes (HFKs) containing wild type HPV16 or CR3 mutant genomes. Cells were grown in monolayer culture and length of time per population doubling was recorded for a maximum of 60 days. (b) Representative Southern blot of episomal viral DNA in wild type HPV16+ cells or integrated viral DNA in HPV16 E7 F57A+ cells. Cell lines made from two HFK donor backgrounds are shown.


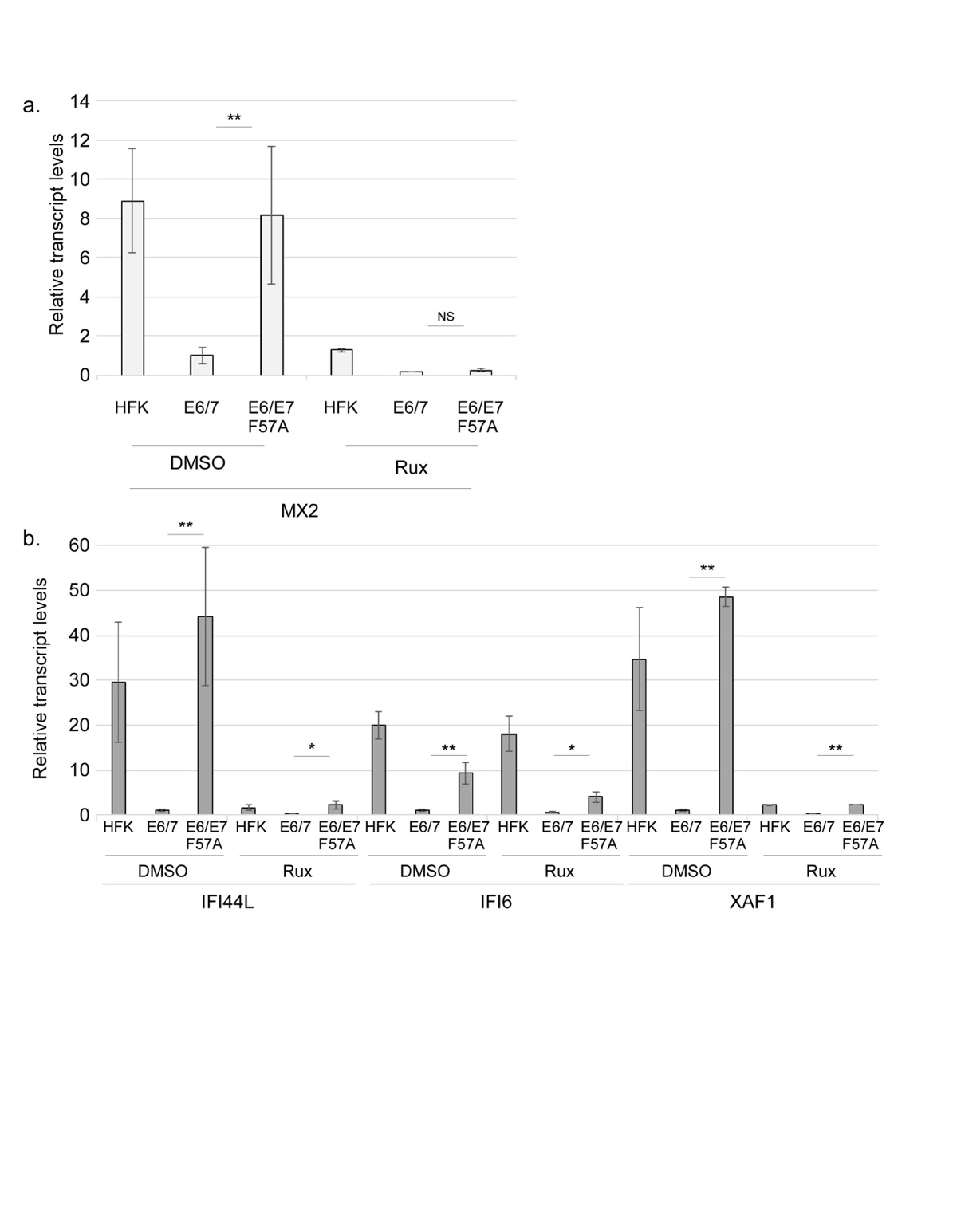
Figure S2: Effect of Ruxolitinib on ISG levels. RT-qPCR analysis of MX2 (a) or IFI44L, IFI6, and XAF1 (b) transcript levels in HFKs, pLXSN E6/E7, and pLXSN E6/E7 F57A cells treated with DMSO (control) or 10 μM Ruxolitinib (Rux) for 24 hours under monolayer culture conditions. Transcripts were normalized to cyclophilin A housekeeping gene, with DMSO-treated pLXSN E6/E7 samples set to 1.


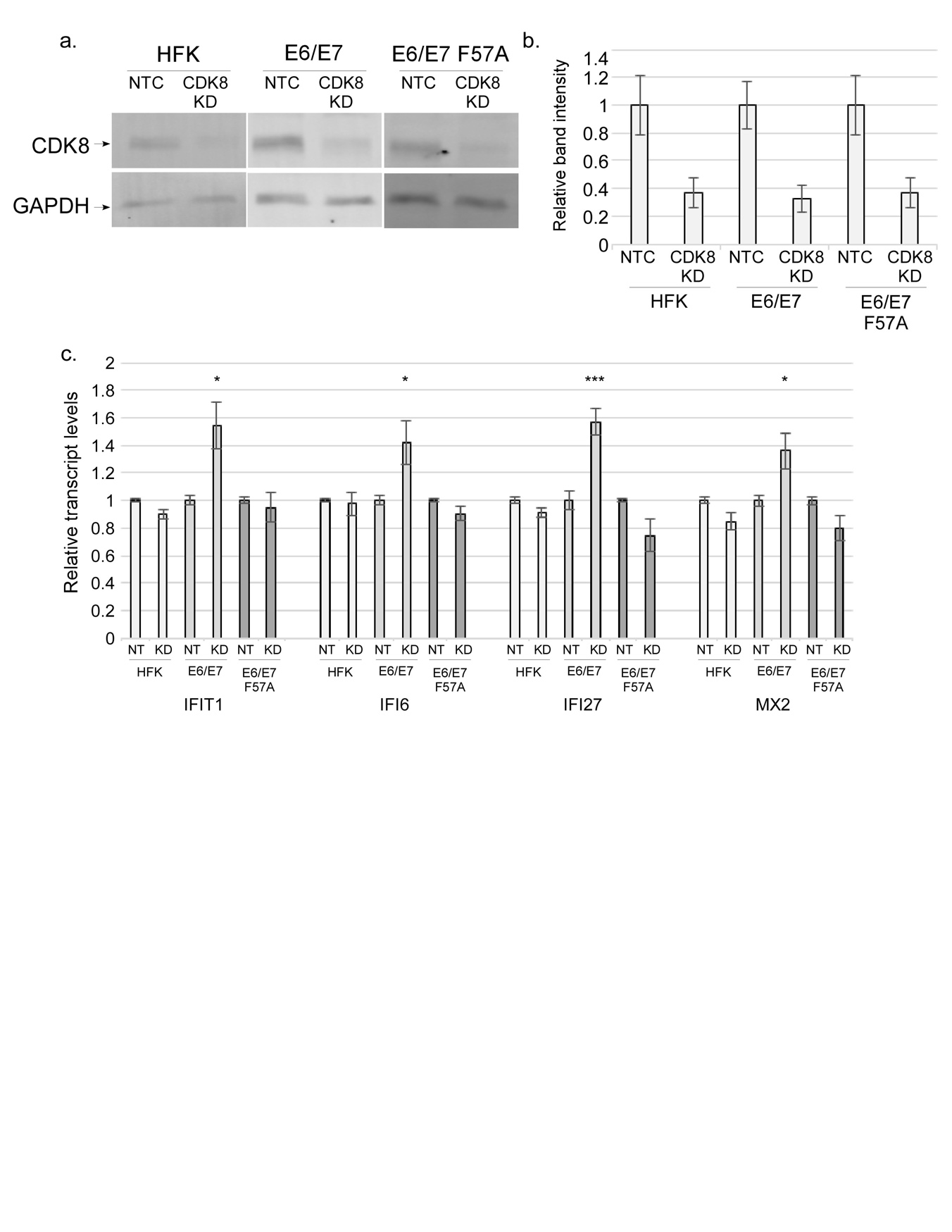


Figure S3: Effect of CDK8 knockdown on ISG expression. (a) Representative western blot of CDK8 and GAPDH protein levels in non-target control (NTC) cells or CDK8 knockdown (CDK8 KD) in the indicated cell types. (b) Quantification of CDK8 knockdown efficiency was determined by normalizing CDK8 band intensity to GAPDH, with NTC samples set to 1. n=3. (c) RT-qPCR analysis of IFIT1, IFI6, IFI27, and MX2 transcript levels in NT or CDK8 KD HFK, pLXSN E6/E7, and pLXSN E6/E7 F57A cells. Transcripts were normalized to cyclophilin A housekeeping gene with NTC values for each cell line set to 1.


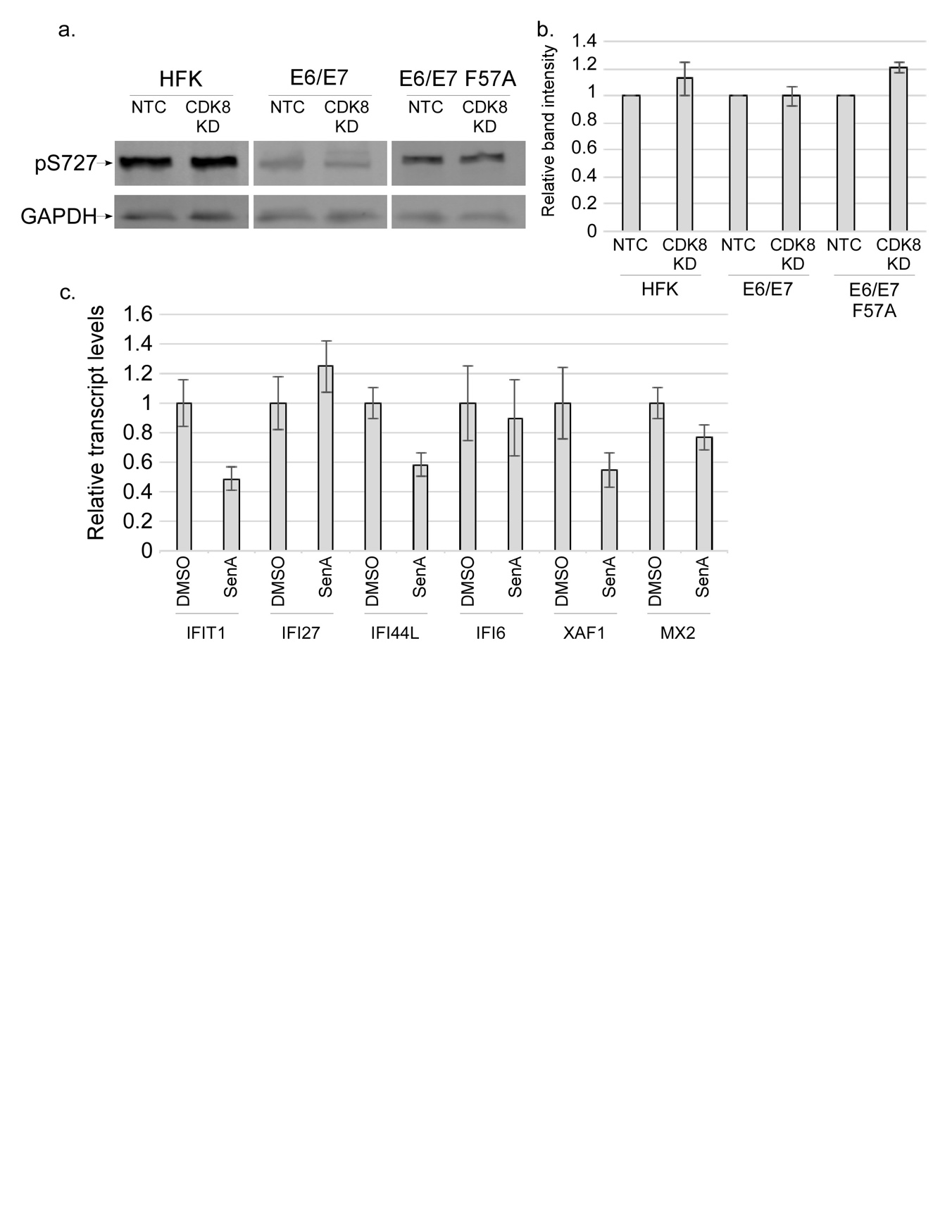
Figure S4: Effect of CDK8 kinase inhibition on ISG expression. (a) Representative western blot of pS727 and GAPDH protein levels in non-target control (NTC) cells or CDK8 knockdown (CDK8 KD). (b) Quantification of pS727 levels was determined by normalizing pS727 band intensity to GAPDH, with NT samples set to 1. n=3. (c) RT-qPCR analysis of IFIT1, IFI27, IFI44L, IFI6, XAF1, and MX2 transcript levels in pLXSN E6/E7 cells that were treated with DMSO (control) or 10 μM Senexin A for 24 hours under monolayer culture conditions. Transcripts were normalized to cyclophilin A housekeeping gene, with DMSO-treated pLXSN E6/E7 samples set to 1.
