# Supplementary figures and images for: Suppression of a Subset of Interferon-Induced Genes by Human Papillomavirus Type 16 E7 via a Cyclin Dependent Kinase 8-Dependent Mechanism
Source: Viruses. 2020 Mar 13;12(3):311. doi: 10.3390/v12030311 (PMC7150855; doi:10.3390/v12030311)

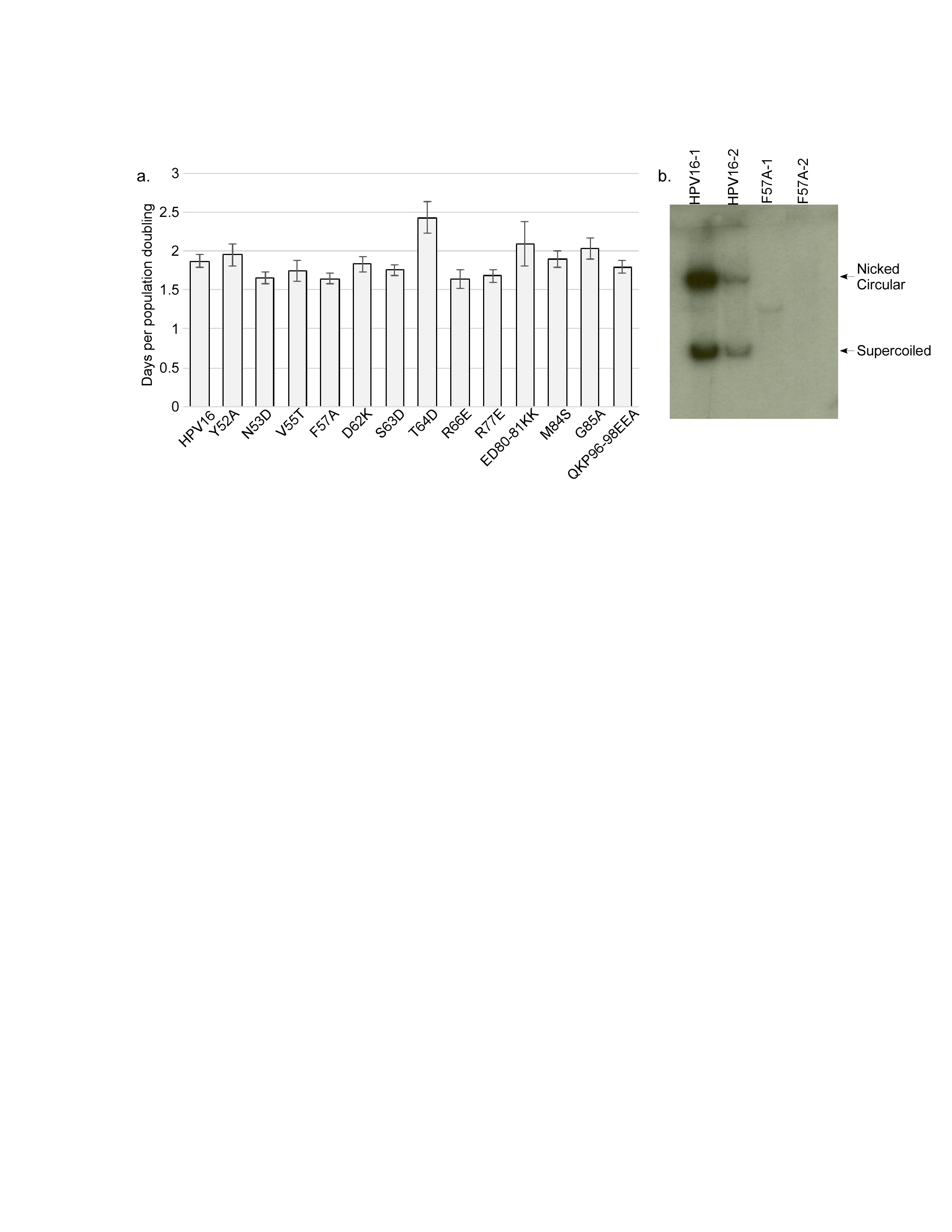

Supplement: Supplementary file 1 [file viruses-12-00311-s001.zip › Supp Figure 1.jpg]

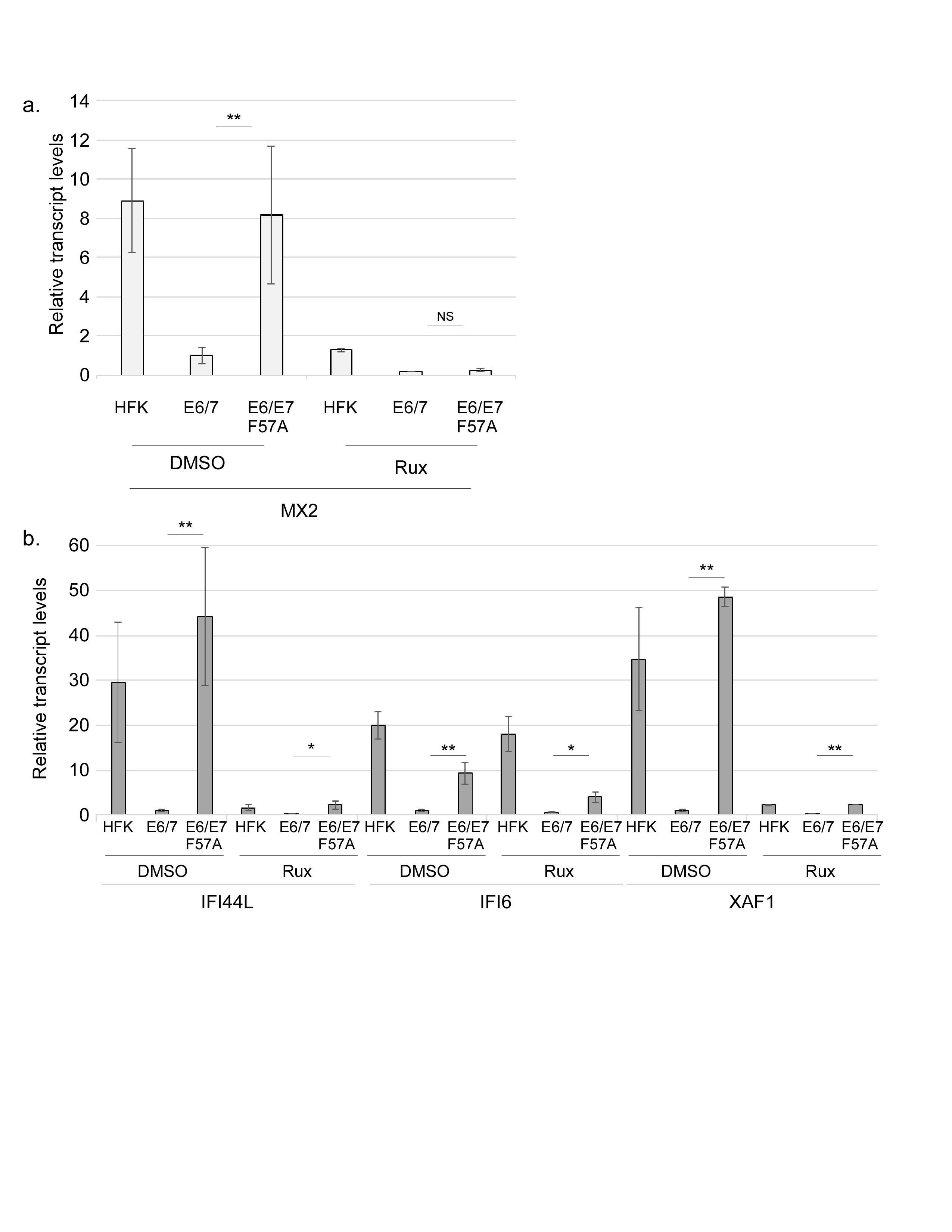

Supplement: Supplementary file 1 [file viruses-12-00311-s001.zip › Supp Figure 2.jpg]

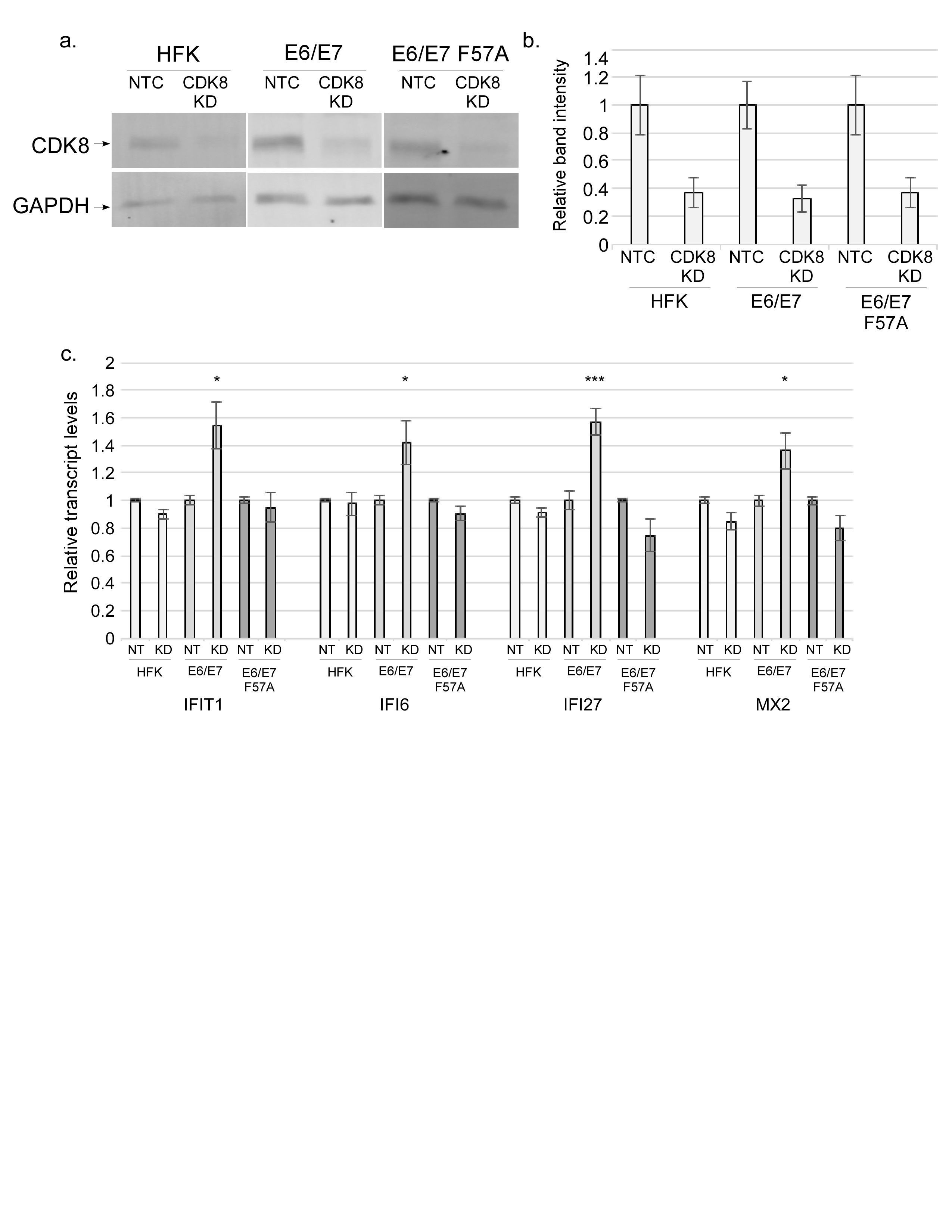

Supplement: Supplementary file 1 [file viruses-12-00311-s001.zip › Supp Figure 3.jpg]

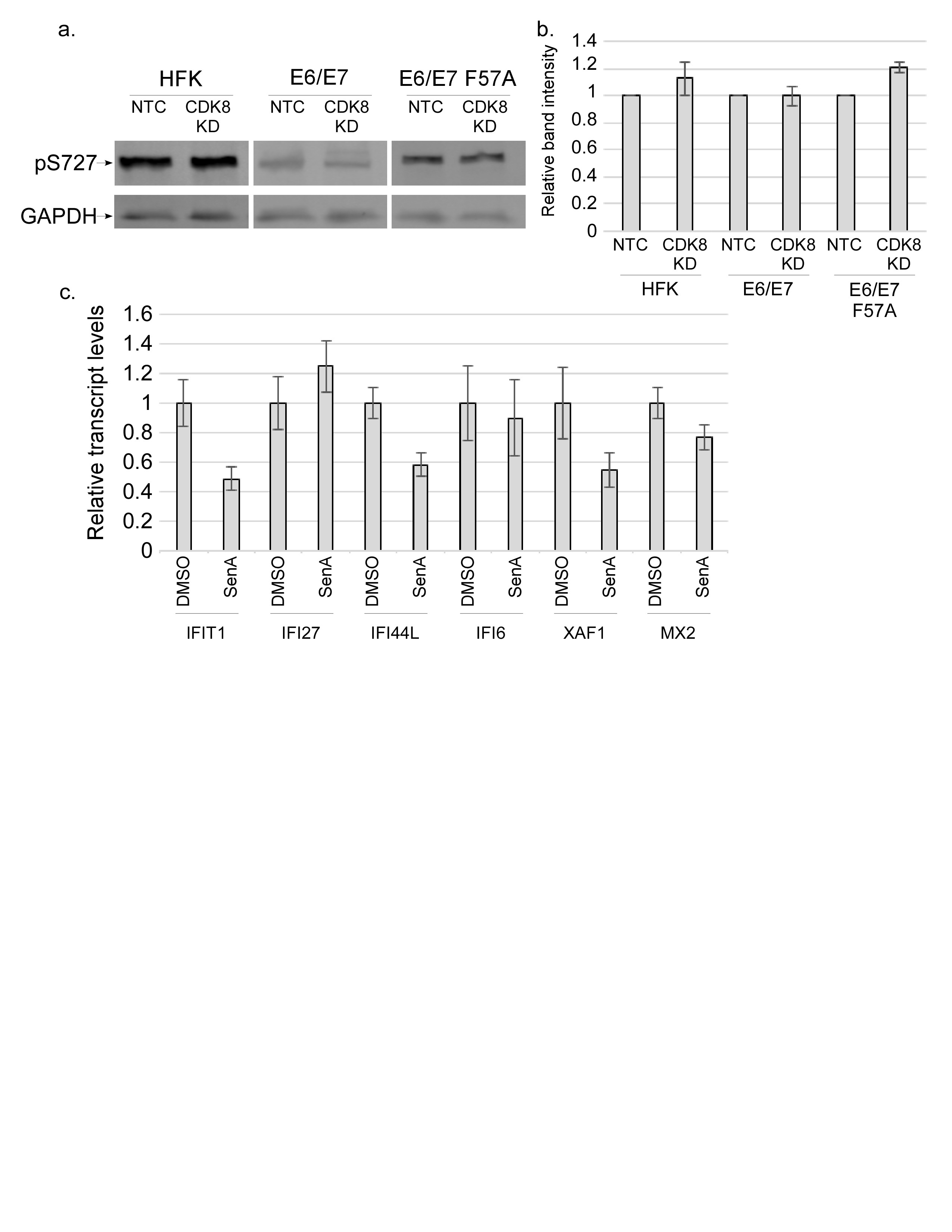

Supplement: Supplementary file 1 [file viruses-12-00311-s001.zip › Supp Figure 4.jpg]
